# DART-3DGS: Distance-Aware Replication and Age-Gated Densification for 3D Gaussian Splatting

**DOI:** 10.3390/jimaging12100483

**Published:** 2026-10-04

**Authors:** Mohannad Adnan Al-Jaafari, Firas Abedi, Qiong Liu, You Yang

**Affiliations:** 1 School of Electronic Information and Communications, Huazhong University of Science and Technology, Wuhan 430074, China; inj.muh@atu.edu.iq (M.A.A.-J.); q.liu@hust.edu.cn (Q.L.); 2 Wuhan National Laboratory for Optoelectronics, Wuhan 430074, China; 3 Najaf Technical Institute, Al-Furat Al-Awsat Technical University, Kufa 31001, Iraq; firasabedi@atu.edu.iq

**Keywords:** novel view synthesis, 3D Gaussian Splatting, initialization, adaptive density control, real-time rendering

## Abstract

3D Gaussian Splatting (3DGS) renders novel views in real time, but quality is limited by two coupled factors: a Structure-from-Motion (SfM) seed that is thin in scene peripheries and weakly textured regions, and an adaptive density-control rule that lets new Gaussians split repeatedly, inflating primitive count, memory, and training time. We address both with two backbone-agnostic add-ons that leave the rasterizer and pruning untouched. Distance-Aware Replication on Tangent-planes (DART) enriches the seed at initialization, replicating each SfM point on its tangent plane within a distance-aware radius, and adding coverage in the scene periphery, where the SfM cloud is typically sparsest, without depth or external cues. Age-Gated Eligibility (AGE) bounds the budget by letting a Gaussian split or clone only after reaching a minimum age, turning the splitting cascade into controlled refinement. Applied unchanged to vanilla 3DGS and anchor-based Scaffold-GS on three standard benchmarks, both add-ons improve both backbones: the Scaffold-GS variant is strongest on six of nine dataset–metric combinations and second on the rest, and under a second protocol leads all three Tanks & Temples metrics and both Deep Blending SSIM/LPIPS, with PSNR within 0.5% of the top result. Gains concentrate on sparsely seeded scenes, while AGE bounds the added cost, lowering primitive count, memory, and training time relative to DART alone at unchanged quality.

## 1. Introduction

Novel view synthesis (NVS) underpins applications from immersive media and aerial imaging [1] to robotics and digital twins. 3D Gaussian Splatting (3DGS) [2] has become the dominant representation for real-time, photorealistic NVS: a scene is encoded as a set of anisotropic 3D Gaussians, *initialized* from a sparse Structure-from-Motion (SfM) point cloud, *grown* by an adaptive density-control (ADC) heuristic that clones and splits Gaussians by a view-space gradient threshold, and rendered by a differentiable tile-based rasterizer. Anchor-based backbones such as Scaffold-GS [3] retain the same two ingredients: an SfM-seeded point set and a growth rule.

The SfM seed, however, is sparse and uneven. Feature matching returns many points on well-textured surfaces near the cameras but few in the scene periphery and in weakly textured regions, so the initialization is dense at the center and thin far away. This deficiency is documented: 3DGS quality has been shown to hinge on the initial cloud, with blur and needle-like artifacts where initial points are inadequate [4], an effect traced to the sparse and incomplete SfM clouds returned in texture-less regions  [5]. Under standard ADC the reconstruction inherits this imbalance: far, sparsely seeded regions recover with visibly less fine detail, while the uniform gradient-threshold split/clone rule drives a densification cascade that inflates the Gaussian count, the peak memory, and the training time. The quality a 3DGS model attains is therefore governed by two coupled but separately addressable factors: *where* coverage is first seeded, and *how* that coverage is allowed to grow.

Most recent work targets the second factor. Refined or principled densification (pixel-aware growth [4], constrained-count representation [6], budgeted densification [7], perceptual allocation [8], and MCMC relocation with a compactness regularizer [9]) improves where and how Gaussians split, yet begins from the same sparse seed. Work that enriches the seed itself is largely learned or depth-based: Gaussian-process densification from pixels and estimated depth [5], or multi-view progressive propagation [10]; the geometric alternative, GeoGaussian [11], aligns Gaussians to SfM normals but depends on reliable normals and is validated on indoor sequences. A depth-free, purely geometric enrichment of the SfM seed that concentrates new coverage in the sparse periphery, needs no learned model and no depth, adds no loss, and transfers across backbones, paired with explicit control of the growth it induces, remains missing.

We propose two components, backbone-agnostic plug-ins that act at two disjoint stages of a point-initialized pipeline; neither component itself modifies the rasterizer or the pruning criteria. Distance-Aware Replication on Tangent-planes (DART) acts once, before optimization: at each SfM point it builds a local tangent frame and replicates the point on a tangent disk whose radius grows with the point’s distance from a robustly estimated scene center, so it adds coverage in the scene periphery, where the SfM cloud is typically sparsest. Unlike GeoGaussian, which reshapes Gaussians and depends on reliable normals, DART uses the tangent plane only to add points; and unlike learned or depth-based enrichment it is deterministic, using neither a trained model nor estimated depth. Age-Gated Eligibility (AGE) acts during optimization: it defers a newly created Gaussian’s first split-or-clone decision until it has matured, so each primitive settles before it is judged. This temporal eligibility lever is distinct from relocation  [9] and from gradient-criterion reweighting [4]; it damps the cascade that DART’s denser seed would otherwise amplify and bounds the final budget. Both are trained under the compactness regularizer of 3D-GS-MCMC [9], which we adopt as an implementation detail rather than a contribution and isolate in our ablation.

**Figure 1:**
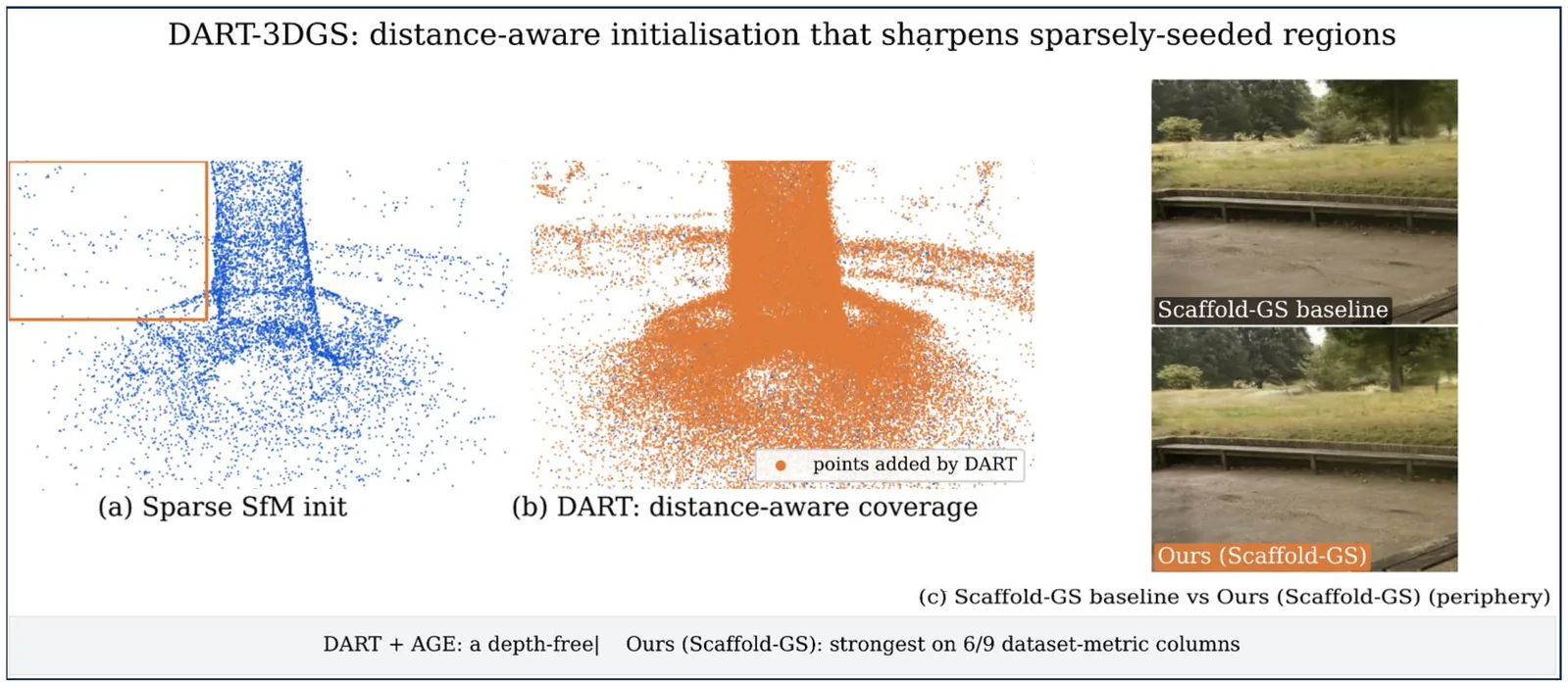
DART in one view. (**a**) The sparse COLMAP SfM cloud that seeds 3D Gaussian Splatting, thin in the scene periphery (blue dots: SfM points; the orange box marks a sparsely seeded peripheral region). (**b**) After Distance-Aware Replication on Tangent-planes (DART), each point is replicated on its local tangent disk with a radius that grows toward the periphery (orange dots: points added by DART; blue dots: original SfM points), so new coverage is concentrated in the periphery, where the seed is typically sparsest; the operation is depth-free and learning-free. (**c**) A peripheral crop of the rendered view comparing the Scaffold-GS baseline with our strongest configuration, Ours (Scaffold-GS), Scaffold-GS with DART and AGE, which recovers sharper detail in the sparsely seeded periphery. The add-on is backbone-agnostic and, paired with Age-Gated Eligibility (AGE) to bound the primitive budget, improves both backbones it is applied to (Section 4).

Figure 1 previews our distance-aware initialization and the sharper far-region detail it produces on a sparsely seeded scene. Applied with identical hyperparameters to both 3DGS [2] and Scaffold-GS [3] on Mip-NeRF 360 [12], Tanks & Temples [13], and Deep Blending [14], the add-ons improve both backbones. Under a like-for-like protocol the Scaffold-GS variant is the single strongest configuration on six of the nine dataset–metric columns, and second on the remaining three. Under a second, independent re-evaluation protocol, it again leads all three Tanks & Temples metrics and both Deep Blending SSIM and LPIPS; on 3DGS, against a protocol-matched reproduction, the add-ons raise Mip-NeRF 360 fidelity by 0.30 dB with consistent SSIM and LPIPS gains. The improvements are largely perceptual and concentrate on the sparsely seeded outdoor scenes, matching DART’s design, while AGE reduces the primitive count, memory, and training time relative to DART alone, at unchanged quality. We make no claim of state-of-the-art peak PSNR against specialized during-training densifiers; our goal is a transferable initialization-and-gating layer that lifts whatever backbone it is added to and is designed to compose with such methods. Consistent with evidence that strong densifiers can be robust to the seed [9], and that the requirement for accurate initialization can itself be relaxed [15] or removed [16], we do not claim initialization is universally decisive; only that, under standard density control, enriching the sparse periphery measurably improves far-region detail.

### Contributions

- DART, a depth-free, geometry-only augmentation of the SfM initialization that replicates each point on its local tangent plane within a disk whose radius grows with distance from the scene center, so that primitives are added in the scene periphery, where the SfM cloud is typically sparsest; it acts once, before optimization, and is agnostic to the rendering backbone.
- AGE, an age-gated densification-eligibility rule that defers a new primitive’s first split-or-clone by one densification cycle; this damps the densification cascade and bounds the primitive budget at no loss of quality, requiring only one age comparison per primitive and no change to the rasterizer or the pruning criteria.
- A controlled, like-for-like evaluation showing that this *initialization-and-gating* pair is backbone-agnostic: applied with identical hyperparameters to both 3DGS [2] and the anchor-based Scaffold-GS [3] under a single protocol, it improves both backbones, and a matched reproduction baseline, a random-seed analysis, and an ablation separate DART’s quality effect from AGE’s cost effect.

## 2. Related Work

### 2.1. 3DGS as the Core Rendering Backbone

Neural Radiance Fields and their successors set the modern standard for photorealistic NVS but rely on slow volumetric ray-marching. For large-scale outdoor scenes, image-based alternatives reduce this cost by selecting a small subset of input views and fusing them with a dual-network blending model [17]. Even recent NeRF–SDF hybrids built specifically for indoor reconstruction still report artifacts from uneven point and weight distribution [18]. 3D Gaussian Splatting [2] consolidated these advances into an explicit representation: a scene is a set of anisotropic 3D Gaussian primitives rendered through a differentiable tile-based rasterizer, which achieves real-time rendering at quality matching the prior state of the art [12]. Its optimization includes an ADC stage that grows Gaussians during training through heuristic clone, split, and prune operations triggered by accumulated view-space positional gradients, starting from a sparse SfM point cloud. We build directly on this framework: DART acts at its initialization and AGE at its densification-eligibility test, while our components leave the rasterizer and the pruning criteria untouched (Section 3.6 states which rasterizer build our experiments use). The representation has since been extended beyond static offline reconstruction, for example to stereo-based semantic SLAM [19], low-light scenes [20], and semantic communication for sparse-view reconstruction [21].

### 2.2. Representation and Kernel Modifications

A first line of work modifies the 3DGS *representation*. Scaffold-GS [3] replaces free Gaussians with voxel-anchored features that spawn view-dependent neural Gaussians, which produces a more structured and more economical model. Mip-Splatting [22] adds a 3D filter that limits each Gaussian’s frequency to what the training views can support and a 2D filter that mimics a physical pixel, together suppressing aliasing across rendering resolutions; 2D-GS [23] replaces volumetric Gaussians with oriented surfels for more accurate surfaces; GES  [24] generalizes the Gaussian kernel to a generalized exponential function for a more compact fit; FreGS [25] matches the rendered and target images in the frequency domain, coarse-to-fine from low to high frequencies, to curb the blur of over-reconstruction; and 3D-HGS  [26] splits each Gaussian by a cutting plane to raise representational capacity at edges. These methods change the kernel, the parameterization, or the regularizer while keeping single-view supervision and heuristic densification intact. Our add-ons are orthogonal: they add nothing to the representation or the rendering path, and transfer across backbones, which we demonstrate by applying them unchanged to both vanilla 3DGS and the anchor-based Scaffold-GS.

### 2.3. Densification Heuristics and Growth Control

A second line refines the ADC heuristic itself. 3D-GS-MCMC [9] reframes growth as Markov-chain Monte-Carlo sampling, replacing cloning with a principled relocation of low-opacity primitives and adding an $L_{1}$ regularizer on opacity and scale to encourage parsimony. Its reported insensitivity to the initial point set follows from that relocation operator: a primitive can be moved to a region that needs it. Standard ADC has no such operator, since clone and split place children only in the neighborhood of their parent, so density cannot migrate into a region the seed never covered. Pixel-GS [4] weights the growth criterion by the number of pixels a Gaussian covers across views, so that under-reconstructed regions grow more readily, and reports directly that primitives in regions with insufficient initializing points fail to grow under the view-averaged gradient criterion; Mini-Splatting [6] first improves the spatial layout of the Gaussians, splitting blurry ones and re-seeding points from rendered depth, and then prunes by each Gaussian’s contribution to a constrained count; Taming-3DGS  [7] densifies toward a target budget for predictable memory and time; Perceptual-GS [8] allocates finer Gaussians to perceptually salient regions through a learned sensitivity signal and a depth-based reinitialization; FastGS  [27] grows and prunes primitives by each Gaussian’s contribution to image quality seen consistently across views, while DashGaussian [28] schedules the rendering resolution from low to high and grows the primitives in step with it, both speeding up convergence without quality-degrading pruning; and AtomGS [29] derives a percentile scale from SfM neighbor distances to constrain small Gaussians and adds an edge-aware normal loss. Among these during-training densifiers, each controls *where*, *how*, and *how many* Gaussians split; none controls *when* a primitive becomes eligible to split. The exponentially ascending gradient threshold of Grubert et al. [30] changes densification over time, but through one global schedule indexed by the training iteration and applied to all Gaussians alike. AGE supplies exactly this missing temporal axis: it gates eligibility by primitive age (detailed in Section 3.3) and is designed to compose with, rather than replace, the methods above. DART, in turn, targets the standard-ADC regime in which density cannot migrate; we make no claim about pipelines that can relocate primitives freely, where a denser seed is expected to matter less.

### 2.4. Seed Initialization and Geometric Enrichment

3DGS is seeded from a sparse SfM cloud whose quality bounds the achievable reconstruction; the seed is thin precisely where feature matching is weak (the periphery and low-texture regions), a deficiency tied to blur and missing detail [4,5] and studied directly by Foroutan et al. [31], who distill initialization structure from a low-cost NeRF and are evaluated under a non-matching protocol. A contrasting line questions this dependence itself: rather than enriching the seed, it relaxes the need for an accurate one, training from a random initialization through a coarse-to-fine optimization schedule [15], or removing SfM/COLMAP from the pipeline altogether by jointly optimizing camera poses and Gaussians [16]. These target the regime in which reliable SfM is unavailable; DART instead operates in the standard regime where an SfM seed exists but is geometrically thin, enriching that seed rather than replacing or bypassing it. Methods that enrich the seed are predominantly learned or depth-based. GP-GS [5] densifies the cloud with a monocular-depth-driven Gaussian Process and prunes high-variance predictions by uncertainty, and is evaluated on its own synthetic and real split; GaussianPro [10] propagates points across views using multi-view consistency, targeting large texture-less driving scenes and reporting primarily on Waymo and a Mip-NeRF 360 subset (it does not evaluate Deep Blending). InstantSplat [32] bypasses SfM entirely: a pre-trained dense-stereo network (DUSt3R/MASt3R) regresses pixel-aligned point maps and recovers camera poses from a few unposed views, then jointly refines the Gaussians and poses in seconds. Outside the 3DGS pipeline specifically, learned point-cloud upsampling networks such as PU-Net-based frameworks [33] address the same sparsity-and-non-uniformity problem through trained models rather than closed-form geometry. The geometric alternative, GeoGaussian [11], parameterizes thin Gaussians aligned to SfM normals, constrains clone and split to the tangent plane, and adds a co-planarity loss, but depends on reliable normals, and is trained and evaluated on the indoor Replica, TUM-RGBD, and ICL-NUIM sequences using PlanarSLAM point clouds rather than COLMAP SfM. DART differs on all three counts. It is depth-free and learning-free, using geometry alone. It uses the tangent plane only as a sampling surface on which to *add* points (it does not reshape Gaussians, constrain densification, or add a geometric loss) and therefore does not require reliable normals. And its disk radius grows with a point’s distance from the scene center, so the new coverage is concentrated in the sparse periphery rather than spread uniformly. Because it touches only the initial cloud, DART is agnostic to the rendering backbone and can in principle be combined with the densification methods of Section 2.3.

## 3. Materials and Methods

### 3.1. Overview

Our method adds two backbone-agnostic components to point-initialized 3D Gaussian Splatting [2]. Both act outside the renderer and the optimizer, so they attach to any pipeline that starts from a point cloud and grows primitives by density control; we use vanilla 3DGS as the primary backbone and apply the same two components, unchanged, to the anchor-based Scaffold-GS [3] in Section 4.5.

The first component, DART, enriches the initial SfM cloud before the backbone sees it: for each point it places a few extra samples on the local tangent plane, with a radius that grows with the point’s distance from the scene center, so the sparsely seeded periphery receives the widest spread (the effect is visible in Section 4.6).

The second component, AGE, addresses the opposite problem. 3DGS re-evaluates every Gaussian for splitting at every density-control step, so a newly created Gaussian can split again immediately, and its children after it, an uncontrolled cascade that inflates the primitive count, peak memory, and training time in already-dense regions. AGE tags each Gaussian with its birth iteration and lets it split or clone only after it has survived a maturity window $T_{\mathrm{mat}}$, so the cascade becomes a controlled refinement. Its role is cost control: it holds the primitive budget bounded at essentially unchanged quality (Section 4.2), which matters because DART’s denser initialization would otherwise enlarge that budget.

**Figure 2:**
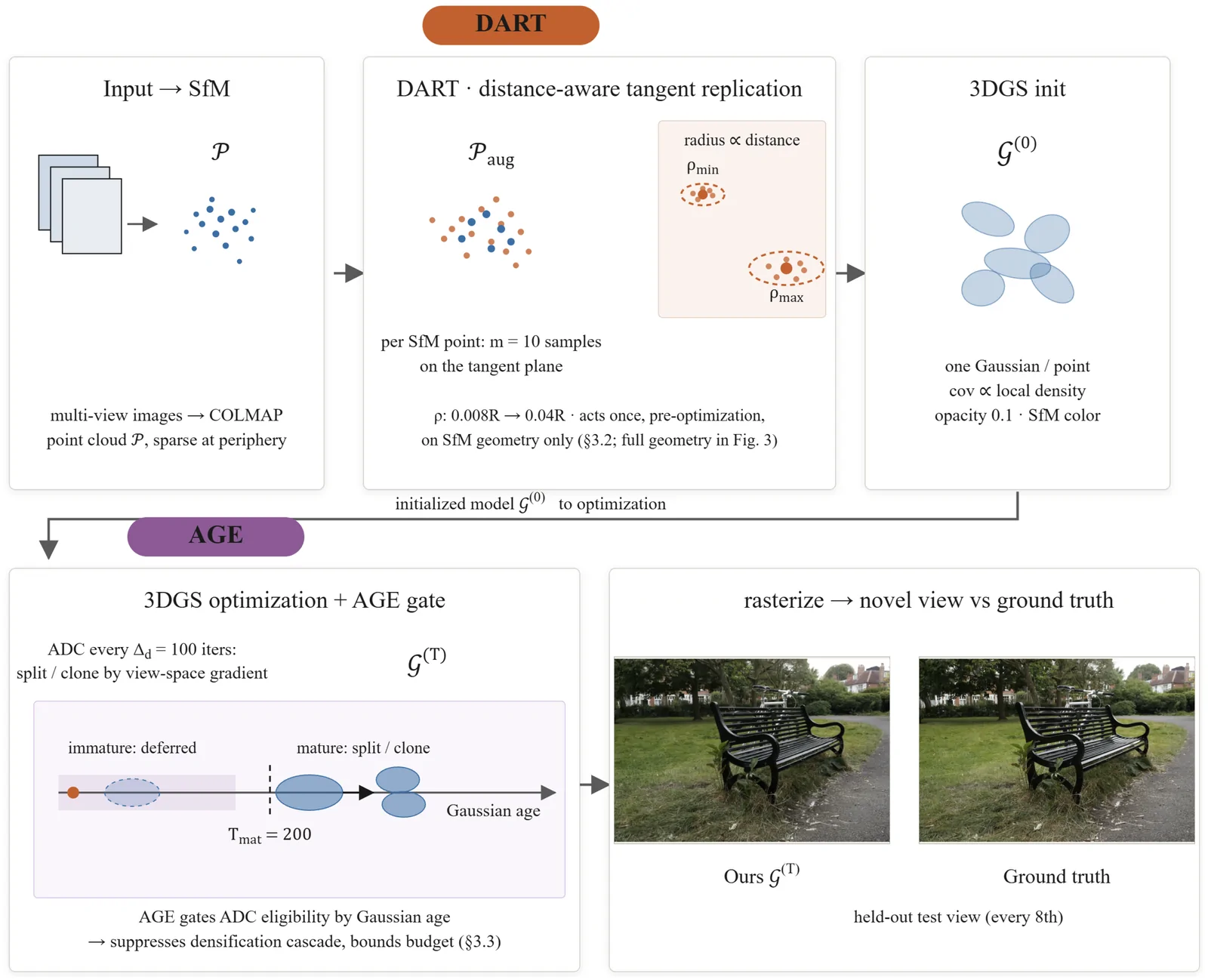
Method overview. Two backbone-agnostic add-ons attach to a standard point-initialized 3DGS pipeline at disjoint stages; by design, neither touches the rasterizer or the pruning criteria. From the sparse SfM cloud P (thin at the periphery), DART (Section 3.2) replicates each point on its local tangent plane within a distance-growing disk, producing the augmented cloud $P_{\mathrm{aug}}$ that initializes $G^{\left(0\right)}$. During training under the standard 3DGS loss with a compactness regularizer (Equation (11)), AGE (Section 3.3) gates densification by primitive age to curb the cascade and bound the budget. The optimized model $G^{\left(T\right)}$ is rendered beside the ground truth. In the figure, § denotes Section and “Fig. 3” refers to Figure 3.

In short, DART sets the starting distribution of primitives and AGE governs how that distribution grows. Figure 2 traces the pipeline: from the SfM cloud P we derive a robust scene scale (Section 3.2), DART produces the augmented cloud $P_{\mathrm{aug}}$ that initializes the Gaussian set $G^{\left(0\right)}$, and training then follows the published 3DGS schedule and rasterizer, modified only by the AGE gate on the densification step. Section 3.2 specifies DART, Section 3.3 specifies AGE, and Section 3.4 details how both integrate into a backbone.

### 3.2. Distance-Aware Replication on Tangent-Planes (DART)

For each SfM point, DART generates m−1 additional samples on the local tangent plane. The sampling disk’s radius grows with the point’s distance from the scene center. Two design choices distinguish DART from existing primitive-growth strategies: it acts once, before optimization, using only the SfM geometry, rather than spawning primitives during training in response to view-space gradients, so it is independent of the backbone’s densification dynamics; and its per-point support is distance-aware rather than uniform, so the sparsely sampled scene periphery receives the widest tangent disks. The construction has four stages: (i) distance, (ii) tangent-plane estimation, (iii) radius assignment, and (iv) disk sampling. We describe each stage below.

*Scene scale.* DART’s radius schedule and per-point distance term both depend on a measure of scene scale. We compute this scale from P alone, before any backbone-specific processing. The scale is summarized by two quantities: a robust center c and a robust radius *R*.

The center c is the L1-median of the cloud,

(1) $$c=\arg\min_{x\in R^{3}}\;\sum_{i=1}^{N}{∥p_{i}-x∥}_{2}.$$

There is no closed-form expression for c, but it is the fixed point of the Weiszfeld iteration,

(2) $$c^{\left(k+1\right)}=\frac{\sum_{i}p_{i}\;/\;∥p_{i}-c^{\left(k\right)}∥}{\sum_{i}1\;/\;∥p_{i}-c^{\left(k\right)}∥}.$$

We initialize $c^{\left(0\right)}$ at the arithmetic mean and run the iteration for $T_{w}$ steps.

The scene radius *R* is the 95th-percentile distance from the scene center,

(3) $$R={\mathrm{Percentile}}_{95}\left(\{∥p_{i}-c{∥}_{2}:p_{i}\in P\}\right).$$

Both c and *R* are computed once before training and held fixed. The L1-median and the percentile radius are standard robust-statistics choices, not contributions of this work; DART consumes the center only through the derived scale, so the exact estimator is not central to the method.

*Stage (i): Distance.* For each $p_{i}\in P$, the distance from the scene center is

(4) $$d_{i}={∥p_{i}-c∥}_{2},$$

and we form the normalized, clipped distance

$${\hat{d}}_{i}=\mathrm{clip}\;\left(d_{i}/R,\;0,\;1\right),$$

where *R* is the 95th-percentile scene radius (Equation (3)). Normalizing by the robust radius *R*, rather than by the maximum distance $\max_{i}d_{i}$, prevents a single distant outlier point from collapsing the radius schedule toward $\rho_{\min}$, and keeps the schedule bounded at the scene periphery.

*Stage (ii): Tangent-plane estimation.* We estimate the local surface normal $n_{i}$ at $p_{i}$ by Principal Component Analysis (PCA) on its $K_{\mathrm{nn}}$ nearest neighbors $N_{i}\subset P$, identified using Euclidean distance in 3D space. $\mathrm{Cov}(N_{i})$ is the 3×3 sample covariance matrix of the 3D coordinates of these neighbors, $\mathrm{Cov}\left(N_{i}\right)=\frac{1}{K_{\mathrm{nn}}}\sum_{j\in N_{i}}\left(p_{j}-{\bar{p}}_{i}\right){\left(p_{j}-{\bar{p}}_{i}\right)}^{⊤}$, where ${\bar{p}}_{i}$ is the centroid of the neighborhood. The normal is the unit eigenvector of $\mathrm{Cov}(N_{i})$ associated with the smallest eigenvalue:

(5) $$n_{i}=\arg\min_{{∥u∥}_{2}=1}\;u^{⊤}\mathrm{Cov}\left(N_{i}\right)\;u.$$

We then complete an orthonormal frame $\left(e_{i}^{\left(1\right)},e_{i}^{\left(2\right)},n_{i}\right)$ at $p_{i}$ by choosing any unit vector $e_{i}^{\left(1\right)}⊥n_{i}$ and setting

(6) $$e_{i}^{\left(2\right)}=n_{i}\times e_{i}^{\left(1\right)}.$$

The pair $\left(e_{i}^{\left(1\right)},e_{i}^{\left(2\right)}\right)$ spans the local tangent plane.

*Stage (iii): Radius assignment.* The disk radius $\rho_{i}$ is a linear function of the normalized, clipped distance ${\hat{d}}_{i}$ (Stage (i)):

(7) $$\rho_{i}=R\cdot \left[\rho_{\min}+{\hat{d}}_{i}\;\left(\rho_{\max}-\rho_{\min}\right)\right],\;{\hat{d}}_{i}=\mathrm{clip}\;\left(d_{i}/R,\;0,\;1\right).$$

Both the bounds $\rho_{\min},\rho_{\max}$ and the distance are expressed relative to the robust radius *R*; this makes the schedule scene-invariant and keeps the radius bounded by $\rho_{\max}R$ even for the farthest points. A point at the scene center receives $\rho_{i}=\rho_{\min}R$; a point at or beyond the 95th-percentile shell receives $\rho_{i}=\rho_{\max}R$.

*Stage (iv): Disk sampling.* For each new sample *k*, we draw two independent uniform variables $\xi_{1},\xi_{2}∼\mathrm{Uniform}\left[0,1\right]$ and map them to polar coordinates on the disk:

(8) $$r=\rho_{i}\sqrt{\xi_{1}},\;\theta =2\pi \;\xi_{2}.$$

The new sample’s 3D position is

(9) $$p_{i}^{\left(k\right)}=p_{i}+r\cos\theta \cdot e_{i}^{\left(1\right)}+r\sin\theta \cdot e_{i}^{\left(2\right)}.$$

**Figure 3:**
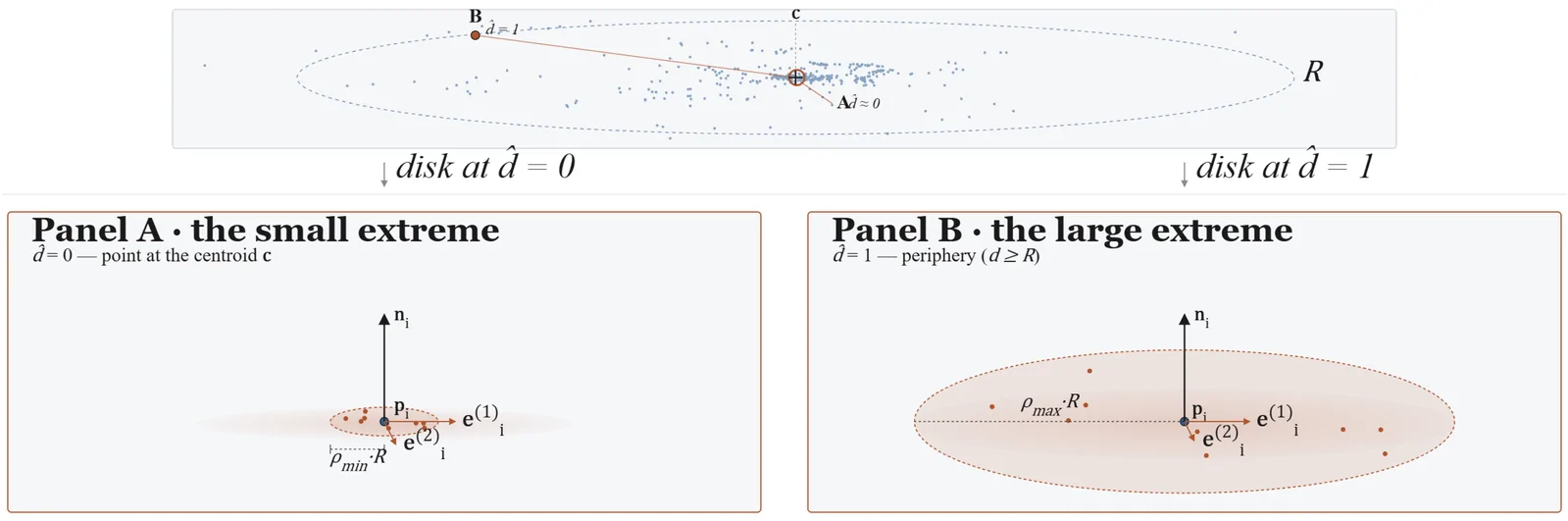
DART geometric construction (Equations (4)–(9)). Before optimization, DART replaces each SfM point $p_{i}$ by m=10 points, the original plus nine replicas drawn uniformly on the local tangent disk, with a radius that grows with the point’s distance from the scene center, so the sparse periphery receives the widest disks. The center c is the cloud’s $L_{1}$-median (Weiszfeld) and *R* its 95th-percentile radius; all lengths are expressed relative to *R*, as the COLMAP scale is arbitrary. The tangent frame $\left(e_{i}^{\left(1\right)},e_{i}^{\left(2\right)}\right)$ is perpendicular to the normal $n_{i}$, the smallest-eigenvalue direction of a local PCA over the $K_{\mathrm{nn}}=20$ nearest neighbors; the disk radius grows linearly with ${\hat{d}}_{i}=\mathrm{clip}\left(d_{i}/R,0,1\right)$, from $\rho_{\min}R$ ($\rho_{\min}=0.008$) to $\rho_{\max}R$ ($\rho_{\max}=0.04$). (**A**): $\hat{d}=0$ (scene center). (**B**): $\hat{d}=1$ (periphery, d≥R), a disk 5× larger; the clip prevents a single distant outlier from collapsing the schedule. Top strip: top-down view of the bicycle scene (Mip-NeRF 360); a 400-point subsample with the horizontal axis stretched ×8.81, so the *R* shell is drawn as an ellipse. Blue dots: SfM points ($p_{i}$ in Panels (**A**,**B**)); orange dots: replicas sampled on the tangent disk; orange markers A and B in the top strip: the two example points.

The new sample inherits the color of its parent point. The PCA normal $n_{i}$ is used only to construct the tangent frame and is not stored on the replica; the backbone re-estimates each Gaussian’s covariance from the augmented cloud and does not consume per-point normals. Algorithm 1 details the full procedure, and Figure 3 illustrates the geometric construction at a single SfM point.

**Algorithm 1** DART: Distance-Aware Replication on Tangent-planes
**Require:** $P=\{p_{i}\}$; multiplier *m*; bounds $\rho_{\min},\rho_{\max}$; PCA neighborhood size $K_{\mathrm{nn}}$; Weiszfeld iterations $T_{w}$**Ensure:** Augmented cloud $P_{\mathrm{aug}}$
1: $c\leftarrow WEISZFELD(P,T_{w})$▹ Equation (2)2: $R\leftarrow {\mathrm{Percentile}}_{95}(\{∥p_{i}-c∥:i\})$▹ Equation (3)3: $P_{\mathrm{aug}}\leftarrow P$4: **for** each $p_{i}\in P$ **do**5:     $d_{i}\leftarrow ∥p_{i}-c∥$▹ Equation (4)6:     ${\hat{d}}_{i}\leftarrow \mathrm{clip}\left(d_{i}/R,0,1\right)$▹ normalized distance, Equation (7)7:     $n_{i}\leftarrow PCANORMAL\left(KNN\left(P,p_{i},K_{\mathrm{nn}}\right)\right)$▹ Equation (5)8:     $\left(e_{i}^{\left(1\right)},e_{i}^{\left(2\right)}\right)\leftarrow TANGENTBASIS\left(n_{i}\right)$▹ Equation (6)9:     $\rho_{i}\leftarrow R\cdot \left[\rho_{\min}+{\hat{d}}_{i}\left(\rho_{\max}-\rho_{\min}\right)\right]$▹ Equation (7)10:     **for** k=1 **to** m−1 **do**11:         $\xi_{1},\xi_{2}∼\mathrm{Uniform}\left[0,1\right]$12:         $r\leftarrow \rho_{i}\sqrt{\xi_{1}}$,   $\theta \leftarrow 2\pi \xi_{2}$▹ Equation (8)13:         $q\leftarrow p_{i}+r\cos\theta \cdot e_{i}^{\left(1\right)}+r\sin\theta \cdot e_{i}^{\left(2\right)}$▹ Equation (9)14:         $q.\mathrm{color}\leftarrow p_{i}.\mathrm{color}$15:         $P_{\mathrm{aug}}\leftarrow P_{\mathrm{aug}}\cup \left\{q\right\}$16:     **end for**17: **end for**18: **return** 
$P_{\mathrm{aug}}$

### 3.3. Age-Gated Eligibility (AGE)

3DGS’s adaptive density control runs a split-or-clone step every $\Delta_{d}$ iterations, duplicating Gaussians whose accumulated 2D-projected positional gradient magnitude exceeds a threshold $\tau_{g}$ (clone for small Gaussians, split for large). At every step the procedure re-evaluates *all* Gaussians, including those born in the immediately preceding step. This permits a cascade: a Gaussian born at step *k* can split at step k+1, its child at step k+2, and so on. In already-dense regions this multiplies primitives faster than the scene needs, which inflates the final Gaussian count, peak memory, and training time and leaves many redundant, under-supported primitives in the set.

*Why age.* The split-or-clone test compares a Gaussian’s view-space positional gradient, accumulated over the preceding densification interval, with $\tau_{g}$. A newly created Gaussian inherits its position and scale from its parent and has no optimizer momentum of its own [30], so during its first interval its gradient mainly reflects its settling away from the inherited state rather than persistent under-reconstruction. Gating eligibility by age lets the unchanged criterion judge each primitive only after this transient.

AGE prevents the cascade by tagging each Gaussian with its birth iteration $b_{i}$ and gating its eligibility for densification on a maturity threshold $T_{\mathrm{mat}}$:

(10) $$\nu_{i}\left(t\right)=t-b_{i},\;\mathrm{Gaussian}\;i\;\mathrm{eligible}\;\mathrm{at}\;\mathrm{iteration}\;t\Leftrightarrow \nu_{i}\left(t\right)\geq T_{\mathrm{mat}}.$$

The mature set $M\left(t\right)=\{i:\nu_{i}\left(t\right)\geq T_{\mathrm{mat}}\}$ proceeds through the standard 3DGS densification criteria (gradient test, scale-based clone-vs-split decision). Immature Gaussians ($\nu_{i}<T_{\mathrm{mat}}$) are skipped at this step and re-checked at the next densification opportunity.

The threshold $T_{\mathrm{mat}}$ controls the maximum cascading rate. With $T_{\mathrm{mat}}=200$ (our default) and the published densification cadence $\Delta_{d}=100$, every Gaussian path can split or clone at most once every two densification cycles. This converts an unbounded cascade into a controlled refinement. The initial set is tagged $b_{i}=0$. Under the published 3DGS schedule, densification begins at iteration $500>T_{\mathrm{mat}}$, so the initial primitives are already mature at the first densification cycle and the gate applies only to primitives created during training; the tag is retained so that AGE remains well-defined under schedules that densify earlier. Because eligibility is gated purely by age, the same maturity test applies to every Gaussian irrespective of origin, SfM-derived or DART-augmented, so AGE acts as a general control on the final primitive budget rather than a mechanism specific to the augmentation (Section 4.2).

### 3.4. Integration into the Backbone

DART and AGE plug into a point-initialized backbone at disjoint stages. We describe the integration for vanilla 3DGS; Section 4.5 applies the identical two components, with the same hyperparameters, to the anchor-based Scaffold-GS without further change. The two stages are:

1. DART rewrites the SfM cloud P into the augmented cloud $P_{\mathrm{aug}}$ (per Section 3.2). The 3DGS initialization then proceeds as published: each augmented point becomes one 3D Gaussian, with covariance estimated from local point density, opacity initialized at 0.1, and color initialized from the SfM color. We denote the initial Gaussian set:

$$G^{\left(0\right)}\;=\;\mathrm{init}\_\mathrm{gaussians}\left(P_{\mathrm{aug}}\right).$$

2. AGE modifies the densification step only (per Section 3.3); it leaves the rasterizer and the pruning criteria untouched (Section 3.6 states which rasterizer build our experiments use).

In every densification cycle, the standard split-or-clone routine is applied, but only to Gaussians in the mature set M(t).

DART and AGE introduce no architectural change and no per-iteration overhead beyond a single age comparison per Gaussian; the two components compose cleanly, with DART controlling the *starting* Gaussian distribution and AGE the *growth dynamics* of that distribution.

*Training objective.* Motivated by the compact-Gaussian regularization of 3D-GS-MCMC [9], which penalizes opacity and scale in $L_{1}$, we optimize the standard 3DGS photometric loss augmented with two lightweight compactness terms. We adopt its opacity term unchanged, but replace the $L_{1}$ penalty on the scale axes with a volume term, the product of the three axes, which penalizes oversized primitives more sharply than their sum:

(11) $$L\;=\;\left(1-\lambda_{D}\right)\;L_{1}\;+\;\lambda_{D}\;L_{D\text{-}\mathrm{SSIM}}\;+\;\lambda_{s}\;\frac{1}{\left|G\right|}\sum_{i}\prod_{j=1}^{3}s_{i,j}\;+\;\lambda_{o}\;\frac{1}{\left|G\right|}\sum_{i}\alpha_{i},$$

where $s_{i,j}$ are the three scale axes and $\alpha_{i}$ the opacity of Gaussian *i*, $\lambda_{D}=0.2$ is the standard D-SSIM weight, and $\lambda_{s}=\lambda_{o}=0.01$. The volume term discourages oversized Gaussians and the opacity term drives redundant Gaussians below the prune threshold, which produces a more compact set. 3DGS^∗^ omits this regularizer, and Section 4.2 isolates its effect.

The single recipe used throughout this paper is therefore:

(12) $$\mathrm{Ours}\;=\;3\mathrm{DGS}\;+\;\mathrm{DART}\;\left(m=10\right)\;+\;\mathrm{AGE}\;\left(T_{\mathrm{mat}}=200\right),$$

and is trained under the compactness-regularized objective of Section 3.4. The default hyperparameters (m=10, $\rho_{\min}=0.008$, $\rho_{\max}=0.04$, $K_{\mathrm{nn}}=20$, $T_{w}=8$, $T_{\mathrm{mat}}=200$) are reported in Section 3.6 and exercised in the ablation of Section 4.2.

### 3.5. Datasets and Evaluation Protocol

*Datasets.* We evaluate on the standard 13-scene benchmark of the 3DGS literature: nine scenes from Mip-NeRF 360 [12] (the outdoor scenes *bicycle*, *flowers*, *garden*, *stump*, *treehill*, and the indoor scenes *room*, *counter*, *kitchen*, *bonsai*) together with two scenes from Tanks & Temples [13] (*truck*, *train*) and two from Deep Blending [14] (*drjohnson*, *playroom*). We use the publicly released images, camera intrinsics, and SfM point clouds. The dataset-standard downsampling is applied: images_4 for the Mip-NeRF 360 outdoor scenes, images_2 for the indoor scenes, and the full-resolution images directory for Tanks & Temples and Deep Blending.

*Evaluation protocol.* We follow the train/test split of Kerbl et al. [2]: every eighth frame is held out for testing, and the remaining frames are used for training. All quantitative results report PSNR, SSIM, and LPIPS [34] measured on the held-out frames at training iteration 30,000. Per-scene scores are averaged within each dataset, and the three datasets are reported separately.

### 3.6. Implementation Details

*Software.* We use the official 3DGS pipeline of Kerbl et al. [2], built on PyTorch 2.0.1 with CUDA 11.8. PSNR and SSIM are computed with the standard implementations bundled with the official evaluation code. LPIPS is computed with the lpips package, version 0.1.4, with the VGG backbone. All experiments use a fixed global random seed of 0; DART’s tangent-disk sampling uses a fixed internal seed of 42, so the augmented initialization is deterministic and identical across runs.

*Hardware.* All experiments, training as well as the time and peak-memory measurements reported in the ablation study (Section 4.2), were run on a single NVIDIA RTX 3070 (16 GB), so all reported timings and memory figures are directly comparable.

*Backbone defaults.* We follow the published default hyperparameters of 3DGS (learning-rate schedule, densification cadence ($\Delta_{d}=100$), gradient threshold $\tau_{g}$, SH degree, and prune cadence) and train for 30,000 iterations. Three engineering settings depart from the 2023 release. First, we use the accelerated rasterizer distributed with the current official 3DGS codebase, which substitutes the drop-in kernels of Taming-3DGS [7]. Second, we train with –optimizer_type sparse_adam, which requires that rasterizer. Third, the D-SSIM term is evaluated with the fused SSIM kernel of the same release. We do not enable –antialiasing, –train_test_exp, or depth regularization, so the train/test split and the evaluation protocol are those of Kerbl et al. [2]. All three substitutions are applied identically to every configuration measured in this paper: the 3DGS^∗^ and Scaffold-GS^∗^ reproductions, every ablation and sweep row, and both Ours variants are produced by a single pipeline, so all comparisons among our own rows are like-for-like by construction. None of the substitutions is part of our contribution; they reduce wall-clock training time, which is what makes the thirteen-scene benchmark tractable on a single GPU. The official release documents a slight quality decrease for the sparse-Adam optimizer relative to the default one, so our reported gains are measured under a marginally conservative configuration rather than an advantageous one. DART and AGE add no change to the rendering path on top of these substitutions. The training objective augments the standard 3DGS photometric loss with the two compactness regularizers of Section 3.4 (a volume term and an opacity term, each weighted $\lambda_{s}=\lambda_{o}=0.01$); the reproduction baseline 3DGS^∗^ (our from-scratch re-run of unmodified 3DGS under this paper’s protocol; Section 3.7) uses the unmodified 3DGS loss.

*DART hyperparameters.* Our method introduces a small set of hyperparameters for the initialization augmentation:

- Replication multiplier m=10.
- Tangent-disk radius bounds $\rho_{\min}=0.008$ and $\rho_{\max}=0.04$ (both as fractions of the scene radius *R*).
- Local-PCA neighborhood $K_{\mathrm{nn}}=20$, i.e., the 20 nearest neighbors of each SfM point under the Euclidean ($L_{2}$) distance between 3D point positions in the SfM world frame.
- Weiszfeld iterations $T_{w}=8$.

The surface normals used to build each tangent frame are estimated by this local PCA ($K_{\mathrm{nn}}=20$) on the SfM cloud; the COLMAP point clouds carry no usable normals of their own, so PCA-estimated normals are used throughout.

*AGE hyperparameter.* The maturity threshold is $T_{\mathrm{mat}}=200$ iterations.

The mathematical definitions of all symbols are given in Section 3 and the Notation.

*Uniform application across scenes.* The configuration above is applied identically to all thirteen scenes; no per-scene hyperparameter tuning is performed.

### 3.7. Compared Methods

We evaluate on the standard 3DGS benchmark, Mip-NeRF 360, Tanks & Temples (truck, train), and Deep Blending (drjohnson, playroom), under the every-8th-image test split and 30,000-iteration budget of Section 3.5 and Section 3.6. Published methods fall into two groups according to the Mip-NeRF 360 scene set they report, and we compute Ours under each group’s protocol so that every comparison is like-for-like.

*Nine-scene protocol (full Mip-NeRF 360; our default).* Reported in Table 1 against the full nine-scene Mip-NeRF 360 set together with the two Tanks & Temples and two Deep Blending scenes: the original 3DGS [2], the anchor-based Scaffold-GS [3], and the densification/schedule methods Mini-Splatting-D [6], Taming-3DGS [7], DashGaussian [28], and FastGS-Big [27]. We re-ran FastGS-Big under our protocol in two settings: with the authors’ released per-scene configurations, and with one unified configuration for all scenes, set to the middle of the authors’ per-scene values (–highfeature_lr 0.005 –grad_abs_thresh 0.0004 –dense 0.001 –mult 0.5); the latter matches our own use of a single configuration throughout.

The original Scaffold-GS paper [3] does not report *flowers* and *treehill*. For those two scenes we substitute our own reproduction under our protocol; the remaining seven Mip-NeRF 360 scenes use the numbers published in that paper.

*Seven-scene protocol (Mip-NeRF 360 minus flowers and treehill).* A second group reports on the seven publicly redistributable Mip-NeRF 360 scenes, omitting flowers and treehill, together with the same Tanks & Temples and Deep Blending scenes: 2D-GS [23], FreGS [25], GES [24], Mip-Splatting [22], 3D-GS-MCMC [9], 3D-HGS [26], and the Scaffold-GS variant of the latter, Scaffold-HGS [26]. To keep these rows on a single protocol, we take evaluation results from the unified re-evaluation of 3D-HGS [26], which re-runs all of them, together with 3DGS and Scaffold-GS, in one pipeline at the published 3DGS training settings. Table 2 reports this comparison, with ours computed on the same seven scenes.

*Reproduction anchor.* Our reproduction of the unmodified 3DGS recipe, denoted 3DGS^∗^; it is the reference row of the ablation in Section 4.2 and the no-augmentation reference against which the sweep of Section 4.3 is read.

*Methods evaluated on different data (not numerically compared).* Several works most related to our initialization question, GaussianPro [10], GP-GS [5], GeoGaussian [11], and the learned-initialization study of Foroutan et al. [31], evaluate on datasets or protocols that do not align with the standard 3DGS benchmark (their evaluation setups are detailed in Section 2.4), so a numerical row in Table 1 and Table 2 would not be like-for-like.

## 4. Results

### 4.1. Main Quantitative Comparison

*Protocol.* We report two matched comparisons. Table 1 uses the full nine-scene Mip-NeRF 360 set with the two Tanks & Temples and two Deep Blending scenes; Table 2 uses the seven-scene Mip-NeRF 360 set (minus flowers and treehill) under the unified 3D-HGS re-evaluation. In both tables, Ours is computed under the same scene set, train/test split, and metric definitions as the competitors beside which it is placed, so each comparison is like-for-like. Per-scene values appear in Table A1, Table A2, Table A3, Table A4 and Table A5 (Appendix A); the contributions of DART and AGE are isolated in Table 3 and Table 4 (Section 4.2 and Section 4.3).

*Headline results.* Our add-on improves both backbones it is applied to. On vanilla 3DGS, Ours (3DGS) is best read against our own 3DGS^∗^ reproduction under the identical protocol, which separates the method’s effect from the protocol gap: plain 3DGS^∗^ already reads 27.38 on Mip-NeRF 360 against the published 27.20. Against 3DGS^∗^, Ours (3DGS) gains +0.30 dB PSNR on Mip-NeRF 360 (27.38→27.68) and improves SSIM and LPIPS on all three benchmarks, with PSNR essentially flat on Tanks & Temples and +0.19 dB on Deep Blending (per-dataset values in Table 1); the gains concentrate on the sparse outdoor scenes (Section 5.2). Both the published 3DGS and our 3DGS^∗^ appear in Table 1. On the anchor-based Scaffold-GS the same two components, unchanged, give Ours (Scaffold-GS), the single strongest row in Table 1. It is best on six of the nine columns and second on the remaining three. Under the seven-scene unified re-evaluation (Table 2), Ours (3DGS) is tied-second on Deep Blending SSIM and mid-table on Mip-NeRF 360.

*Per-dataset summary.* Among the competing methods, our 3DGS variant is competitive rather than leading, and its strengths are perceptual. On Mip-NeRF 360 its PSNR (27.68) is mid-table, while its LPIPS (0.203) is third-best, behind only Mini-Splatting-D (0.176) and our own Scaffold-GS variant (0.193). On Tanks & Temples it is second on SSIM (0.859, behind only our Scaffold-GS variant), but its PSNR (23.65) is among the lowest in the table, about 0.8 dB below the best (24.46) and essentially level with the 3DGS^∗^ baseline. On Deep Blending it is third on SSIM (0.910, tied with Taming-3DGS) and mid-table on PSNR (29.95). Measured against the matched 3DGS^∗^ baseline, the add-on improves or matches every metric except Tanks & Temples PSNR, where it dips a negligible 0.04 dB, and its clearest and most consistent gains are on the perceptual metric LPIPS.

*Where we trail.* The columns where our 3DGS variant is not among the top two are mainly the PSNR scores, most visibly on Mip-NeRF 360 (27.68, fifth) and Tanks & Temples (23.65, among the lowest). The competitors ahead on PSNR on both datasets are FastGS-Big, Taming-3DGS, and DashGaussian, which all act *during* training by changing the densification criterion, schedule, or rendering resolution. DART and AGE act at a different stage from those methods: DART once on the initial point cloud and AGE only as a maturity gate on densification (Section 5.1). Applied to Scaffold-GS, the same DART + AGE raises it past all three on exactly these PSNR columns, reaching 28.07 on Mip-NeRF 360 and 24.46 on Tanks & Temples, against 27.83/24.42 (FastGS-Big^∗^ with per-scene configurations), 27.78/24.06 (Taming-3DGS), and 27.73/24.00 (DashGaussian) (Section 4.5). Cross-dataset patterns are discussed in Section 5.

*Seven-scene comparison.* Table 2 reports a second comparison whose competitor rows are taken from the published 3D-HGS paper [26], where a family of methods is measured on the seven publicly redistributable Mip-NeRF 360 scenes; we insert our two variants into that table.

Because both the data source and the Mip-NeRF 360 scene set differ between the tables (seven scenes here versus nine in Table 1), the absolute numbers are not comparable cell-by-cell, and each table should be read on its own. The two Ours rows are the same trained models in both tables, so their Tanks & Temples and Deep Blending columns are identical; only the Mip-NeRF 360 scene set and the set of competitors change. This is also why the shared vanilla-3DGS row reads 28.88 here (seven scenes) against 27.20 in Table 1 (nine scenes). In this table, Ours (3DGS) is second-best on Tanks & Temples LPIPS (0.154) and Deep Blending SSIM (0.910, tied with Scaffold-HGS), and mid-table on the remaining columns; Ours (Scaffold-GS) is best or second in every column, leading all three Tanks & Temples metrics and both Deep Blending SSIM and LPIPS.

*Read jointly.* Four columns behave the same under both protocols: Ours (Scaffold-GS) is best on all three Tanks & Temples metrics and on Deep Blending SSIM, independent of the scene set or the competitor set. Its PSNR is strong throughout, best on all three datasets in Table 1, and in Table 2 within 0.47% of the top result (3D-GS-MCMC) on Mip-NeRF 360 and within 0.26% of the top (Scaffold-HGS) on Deep Blending; no PSNR value is more than 0.5% below the leader. The only two columns it never leads are Mip-NeRF 360 SSIM and LPIPS, where it is a close second to perceptual-specialized methods: Mini-Splatting-D in Table 1, and 3D-GS-MCMC (SSIM) and Mip-Splatting (LPIPS) in Table 2. On Deep Blending LPIPS it leads in Table 2 but is edged by Mini-Splatting-D in Table 1.

### 4.2. Ablation: Isolating the Contributions of DART and AGE

*Protocol.* We isolate each component with a controlled ablation over four scenes spanning the three dataset families: *bicycle*, *stump* (Mip-NeRF 360 outdoor), *counter* (Mip-NeRF 360 indoor), and *drjohnson* (Deep Blending); means reported in this subsection are over these four scenes and therefore differ from the nine-scene Mip-NeRF 360 aggregates of Table 1. The configurations are:

1. 3DGS^∗^: our reproduction of the published 3DGS recipe with the unmodified loss (baseline).
2. 3DGS^∗^ + Reg: adds the compactness regularizer of Section 3.4 (volume + opacity), which isolates its effect before any of our components.
3. 3DGS^∗^ + Reg + DART (m=1) + AGE: AGE with DART disabled, since m=1 draws no extra samples ($P_{\mathrm{aug}}=P$); read against configuration 2 this isolates AGE on the un-augmented cloud.
4. 3DGS^∗^ + Reg + DART (m=10): adds the DART augmentation with AGE off; read against configuration 2 this isolates DART.
5. 3DGS^∗^ + DART (m=10) + AGE (= Ours without Reg): the full DART + AGE model with the regularizer removed.
6. 3DGS^∗^ + Reg + DART + AGE (= Ours): the complete method, applying the AGE maturity gate ($T_{\mathrm{mat}}=200$) to the augmented, regularized model; read against configurations 4 and 5 it isolates AGE and the regularizer, respectively, at the full model.

*Geometry controls.* Configurations 7–9 are of a different kind: rather than adding a component, each replaces a single geometric design choice inside configuration 6, holding the replica count (m=10), the sampling seed, and the training objective fixed. Configurations 7 and 8 remove the distance-awareness by fixing the radius at ρ=0.024, the midpoint of the schedule, and at $\rho =\rho_{\max}=0.040$, its upper bound; the midpoint variant preserves the mean added spread and changes only its spatial distribution, so it tests the schedule rather than the amount of added coverage. Configuration 9 keeps the distance-aware radius but draws replicas uniformly inside an isotropic ball rather than on the tangent disk. Read against configuration 6, these isolate the two geometric assumptions named in the method.

Together these isolate each component with the others held fixed: the regularizer against the baseline (1 vs. 2) and at the full model (5 vs. 6); DART with the regularizer on and AGE off (2 vs. 4); and AGE with the regularizer fixed, both without DART’s extra points (2 vs. 3) and with them (4 vs. 6); the geometry controls are read against the complete model (6 vs. 7–9). Table 3 reports the quality metrics and the final Gaussian count for all nine configurations.

*Are the two geometric choices load-bearing?* All three controls lose against the complete method. The uniform upper-bound radius is the most damaging: at 26.25 dB it falls 1.28 dB *below* the no-replication baseline of configuration 2, and on *stump* it collapses by 6.0 dB while its final primitive count drops to 2.2 M after a peak allocation of 14.0 GB, indicating that replicas placed too far from the surface inflate the model early and are then pruned away. Replication with the wrong radius is therefore worse than no replication at all, which indicates that primitive count alone does not explain our gains. The isotropic ball reaches the same conclusion from the opposite direction: it loses quality while producing 11.7%*more* primitives than the complete method (3.52 M versus 3.15 M).

The three substitutions stress different scenes. The constant-midpoint radius costs 0.22 dB on *stump* and 0.21 dB on *drjohnson*; the isotropic ball costs 0.42 dB on *stump*; and the constant-upper-bound radius degrades *bicycle* and *counter* as well, raising their LPIPS by 0.018 and 0.007 against a seed-induced variation of $2\times 10^{-4}$ (Section 4.4).

Every scene in the ablation set is materially affected by at least one substitution, and the upper-bound variant degrades SSIM and LPIPS on all four scenes, so the result does not rest on a single scene. The pattern matches the intent of the schedule: a small $\rho_{\min}$ protects the densely seeded interior while a large $\rho_{\max}$ serves the sparse periphery, and no single constant achieves both.

*Practical guidance.* The schedule needs no adjustment to scene scale: $\rho_{\min}$ and $\rho_{\max}$ are fractions of the robust radius *R*, so the arbitrary global scale of a COLMAP reconstruction cancels out. Nor does it need adjustment to SfM density: the median nearest-neighbor spacing of the seed, relative to *R*, varies about eightfold across our thirteen scenes (from $4.2\times 10^{-4}$ on *truck* to $3.4\times 10^{-3}$ on *playroom*), yet the single default schedule improves SSIM and LPIPS on all thirteen (Table A3 and Table A5). The failure of configuration 8 does not come from a mis-set endpoint but from removing the distance dependence, i.e., giving every point the largest disk. We therefore recommend keeping the default schedule, never enlarging the radius uniformly to compensate for sparsity, and lowering *m* rather than changing ρ for densely reconstructed scenes (Section 4.3).

*Contribution of the regularizer.* The compactness regularizer is an adopted training detail (Section 3.4, following 3D-GS-MCMC [9]), not a component we propose; we report its effect only to confirm that including it is not detrimental. On the full model, adding it (Ours without Reg → Ours) raises PSNR by 0.05 dB (to 27.80) and reduces the final Gaussian count by 2.5% (3.23→3.15M, Table 3), while SSIM improves by 0.001 and LPIPS worsens by only 0.001 (0.203→0.204), a negligible perceptual cost. The effect runs in the same direction at the baseline (+0.08 dB, −2.3% count). Inclusion is therefore a small net positive, marginally higher fidelity at a slightly smaller budget, which is why we retain it; it is reported in isolation so that it is not conflated with the contributions of DART and AGE.

*Disentangling the regularizer from DART and AGE.* Configurations 1, 2, 5, and 6 form a complete 2×2 factorial design over the regularizer (off/on) and DART + AGE (off/on). The effect of each factor is nearly unchanged by the state of the other: DART + AGE raises PSNR by 0.30 dB without the regularizer (1→5) and by 0.27 dB with it (2→6), with the same SSIM gain (+0.014) and LPIPS reduction (0.025 and 0.024) in both cases, whereas the regularizer adds 0.08 dB without DART + AGE (1→2) and 0.05 dB with them (5→6), changing SSIM and LPIPS by at most 0.001. The two factors therefore do not confound each other: DART + AGE account for about 86% of the PSNR gain of the complete model and essentially all of its SSIM and LPIPS gains, while the regularizer contributes a small, separable increment. Within DART + AGE, the remaining configurations separate the two components with the regularizer held fixed: DART supplies the quality gain (2→4) and AGE the cost reduction (2→3 and 4→6).

*AGE without DART augmentation.* Configuration 3 (m=1, the gate with no DART augmentation) read against the regularizer-only row isolates AGE when DART adds no points: it lowers the final Gaussian count from 2.91 to 2.63M (a 9.6% reduction, Table 3) with quality essentially unchanged (PSNR +0.04 dB, SSIM unchanged, LPIPS +0.002). AGE therefore removes close to a tenth of the primitives even on the raw, un-augmented cloud, so its effect does not depend on DART’s extra samples; the m=10 row read against Ours below quantifies the same effect on top of the augmented cloud.

**Figure 4:**
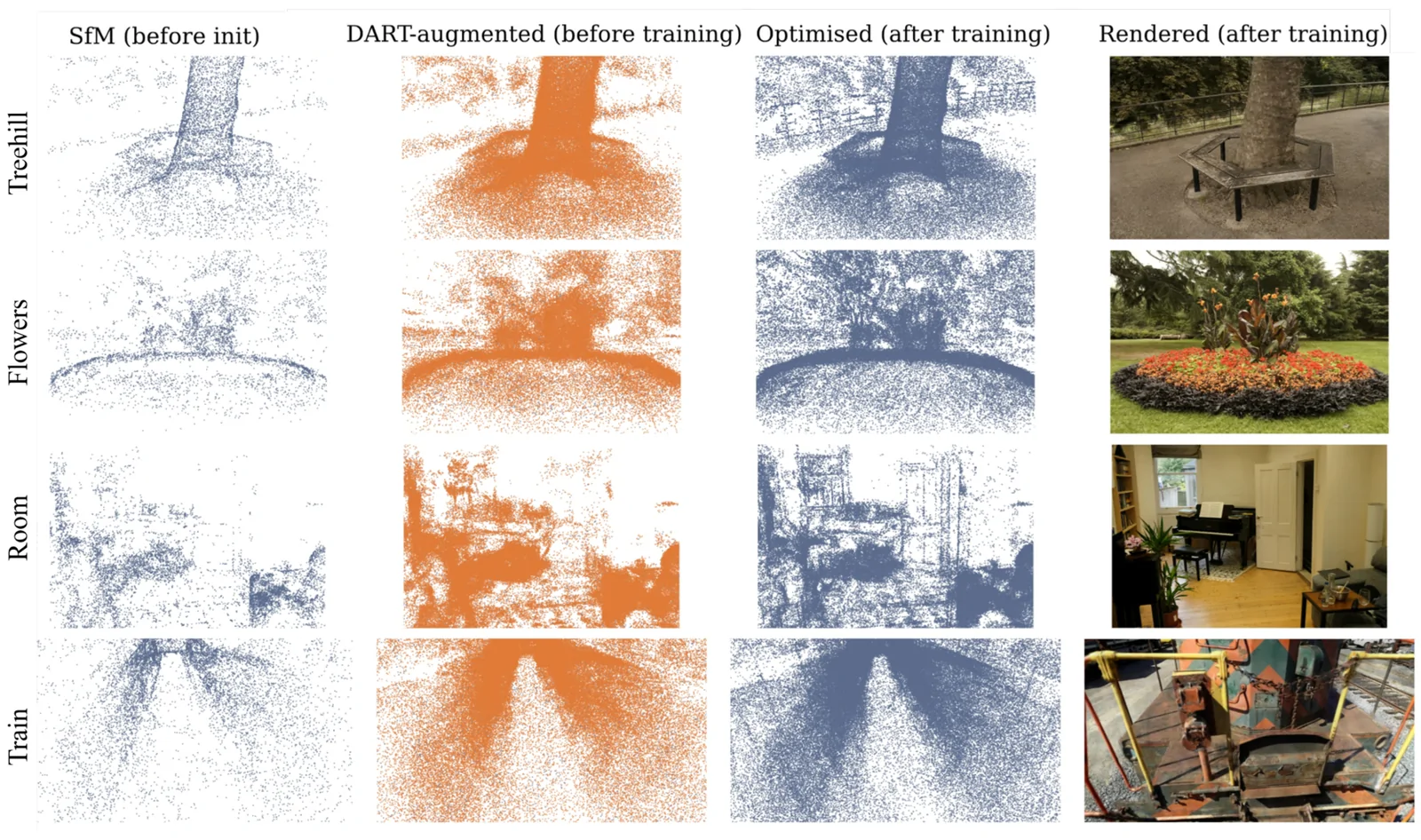
Spatial effect of DART. Rows: *flowers* (Mip-NeRF 360 outdoor), *room* (Mip-NeRF 360 indoor), and *train* (Tanks & Temples). Each row shows, from left to right: the raw COLMAP SfM cloud (before initialization); the DART-augmented cloud that seeds optimization, with the original points and the added tangent-disk samples; the optimized primitive centers (after training); and the rendered view (after training). The three point-cloud panels are projected from the same camera as the rendered view, so their angle matches the render. DART draws m=10 tangent-disk samples per point, concentrated in the sparsely reconstructed periphery.

*Contribution of DART.* Adding DART at m=10 on top of the regularizer (configurations 2 → 4) is the source of the quality gain: PSNR rises by 0.22 dB (27.53→27.75), SSIM by 0.013 (0.834→0.847), and LPIPS falls by 0.025 (0.228→0.203; Table 3). The gain is largest on the sparsely seeded Mip-NeRF 360 scenes: LPIPS falls 0.044 on *bicycle* and 0.024–0.026 on *counter* and *stump*, whereas the densely reconstructed Deep Blending scene *drjohnson* is essentially unchanged (Table A9), consistent with DART adding coverage only where SfM points are sparse. This denser initialization enlarges the final set by 18.6% (2.91→3.45 M) and raises training time and peak memory (24.9→31.8 min, 5.04→6.04 GB). DART thus delivers the fidelity gain at the cost of a larger primitive budget, which AGE then trims. Figure 4 shows this spatially across four representative scenes.

*Contribution of AGE on top of DART.* With the regularizer and DART held fixed, enabling AGE (configuration 4 to Ours) reduces the final Gaussian count from 3.45 to 3.15 M (an 8.7% reduction) and lowers training time by 2.4 min (31.8 to 29.4) and peak memory by 0.47 GB (6.04 to 5.57), while quality is unchanged within measurement noise (PSNR +0.05 dB, SSIM +0.001, LPIPS +0.001, Table 3). This 8.7% reduction is close to the 9.6% obtained without augmentation above, so AGE removes a comparable fraction of the cloud whether or not DART has densified it.

**Figure 5:**
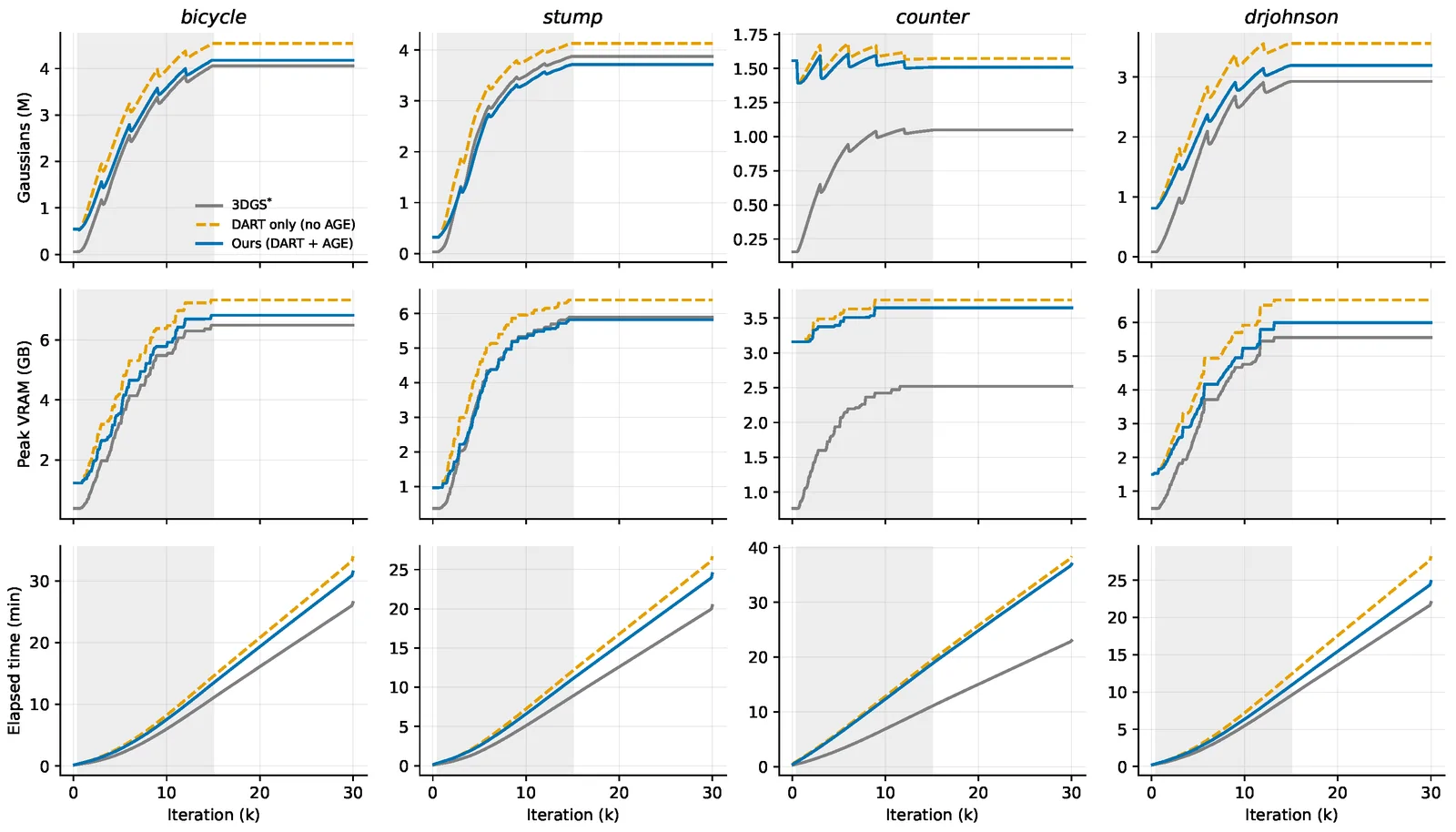
Training cost over iterations on the four ablation scenes for 3DGS^∗^, DART alone (configuration 4), and Ours (configuration 6): Gaussian count (**top**), peak allocated VRAM (**middle**), and elapsed training time (**bottom**). The gray shaded band marks the densification phase (iterations 500–15,000).

Figure 5 locates this effect in time. AGE lowers the Gaussian count during densification, by 16–23% relative to DART alone at iteration 5k on *bicycle*, *stump*, and *drjohnson*, and the peak memory, reached at the end of densification, follows the count. The added cost is therefore not an early-training overhead: the first 500 iterations take at most one minute in every scene.

*Quality is not merely a larger budget.* The fidelity gain is not explained by simply retaining more primitives. Ours reaches its quality at 3.15M Gaussians, only 5.7% above the 2.98M of the baseline (Table 3), and the DART (m=1) + AGE configuration matches or exceeds baseline quality with *fewer* primitives than the baseline (2.63M, at PSNR 27.57 versus 27.45). The improvement therefore reflects better-placed coverage from the distance-aware augmentation, not a larger primitive count.

*Mechanism interpretation.* Taken together, the two regimes reframe AGE’s role. Its count reduction is essentially the same with and without DART augmentation (8.7% versus 9.6%), so the gate is not acting specifically on the extra primitives that DART introduces; rather, by deferring each Gaussian’s eligibility for split-or-clone until it has matured through the maturity window (Section 3.3), it suppresses the persistently under-supported primitives that the unconstrained density-control cascade would otherwise retain, independently of how the initial cloud was formed. AGE is therefore a cost-control gate: deferring immature splits keeps the budget, and the memory and time DART would otherwise inflate, bounded at flat quality. DART supplies coverage and fidelity; AGE controls count.

### 4.3. Hyperparameter Sweep

*Protocol.* Our method introduces two hyperparameters beyond the 3DGS defaults: the DART replication multiplier *m* and the AGE maturity threshold $T_{\mathrm{mat}}$. We sweep *m* on the four ablation scenes of Section 4.2, holding AGE at its default; $T_{\mathrm{mat}}$ is fixed by design rather than swept, for the reason given below.

*Sweep of m (DART multiplier).* We sweep m∈{1,3,5,7,10} with AGE fixed at $T_{\mathrm{mat}}=200$, where m=1 leaves the SfM cloud unaugmented (DART draws m−1=0 extra samples). All other DART hyperparameters ($\rho_{\min}=0.008$, $\rho_{\max}=0.04$, $K_{\mathrm{nn}}=20$) are held at the single set of main-run values used for every scene and dataset; these were fixed once through extensive preliminary experimentation across several distinct scenes, rather than tuned per scene or chosen arbitrarily; Section 4.2 further shows that replacing the schedule by either of its endpoints held constant is worse than the schedule itself, so the gain follows from the distance dependence rather than from the specific endpoint values.

Table 4 reports the full per-scene and per-metric results.

*Choice of $T_{\mathrm{mat}}$ (AGE threshold).* We fix $T_{\mathrm{mat}}=2\Delta_{d}$ by design rather than sweeping it: with the published densification cadence $\Delta_{d}=100$ (so $T_{\mathrm{mat}}=200$), this defers each newly created Gaussian by a full densification interval, so it settles into place before its first split-or-clone decision. We do not tune it per scene.

*Justification of the multiplier.* The quality trend across the sweep favors the largest setting. PSNR saturates early (it rises 0.21 dB from m=1 to m=3, from 27.57 to 27.78, and is essentially flat thereafter, gaining only 0.02 dB more through m=10), but the perceptual and structural metrics keep improving monotonically in the mean: LPIPS falls from 0.230 at m=1 to 0.204 at m=10, and SSIM rises from 0.834 to 0.848 (Table 4). This mean trend is driven by the large, sparsely reconstructed outdoor scenes; the augmentation helps these rather than the dense indoor ones, and the dense indoor scene drjohnson does not benefit from heavy replication and slightly regresses past m=3 (PSNR 29.58→29.31), consistent with DART adding coverage only where the SfM cloud is sparse.

Because DART targets perceptual coverage rather than peak PSNR, we adopt the setting that maximizes these metrics, m=10. By the mean alone, m=7 already attains almost the same quality with a smaller initial cloud (LPIPS within 0.003, SSIM within 0.001), so m=10 is not uniformly optimal; rather, we fix a single conservative value that targets the sparse outdoor scenes most generally, while a smaller *m* may be chosen for dense indoor scenes, which would further improve the cost–quality trade-off. We retain m=10 for two reasons. First, several scenes still gain appreciably at this setting, well beyond the mean: bicycle LPIPS improves 0.006 and counter PSNR rises 0.06 from m=7 to m=10 (versus 0.003 and 0.01 in the mean). Second, reporting m=10 characterizes the method at its heaviest setting rather than a relaxed one, so the primitive count, memory, and time we report reflect a worst case, even though a lighter *m* remains a viable option.

### 4.4. Robustness to Random Seed

A small mean improvement invites the objection that it could be an artifact of a single lucky random seed. We therefore quantify the run-to-run variation of both the baseline and our method and compare it against the size of the gain. We retrain the unmodified 3DGS^∗^ baseline and our full method (DART + AGE, under the compactness-regularized objective of Section 3.4) with three global training seeds {0,1,2} on two representative scenes, *bicycle* (large outdoor, sparse SfM) and *counter* (indoor, dense SfM). DART’s internal tangent-disk sampling seed is held fixed at 42 throughout, so the augmented initialization is identical across the three runs and the measured variation reflects the backbone’s stochastic optimization alone. Table 5 reports the per-scene mean ± standard deviation across the three seeds.

The per-scene run-to-run standard deviation is at most 0.019 dB (PSNR), 0.0005 (SSIM), and 0.0008 (LPIPS), one to two orders of magnitude below the corresponding improvements over the baseline (+0.32 dB/+0.026/−0.042 on *bicycle*; +0.27 dB/+0.008/−0.023 on *counter*). Dividing each improvement by the larger of the two relevant standard deviations, every gain exceeds its seed-induced variation by more than an order of magnitude, from 14× at the tightest (counter PSNR) to 113× at the widest (counter LPIPS); the reported gains are therefore not attributable to seed selection.

### 4.5. Composability with Scaffold-GS

DART and AGE are backbone-agnostic add-ons: DART augments the SfM cloud before optimization, and AGE gates primitive growth during it, with neither touching the rendering path or the optimizer. To test this directly, we apply the same two components, with the identical hyperparameters used on 3DGS (m=10, selected in Section 4.3; $T_{\mathrm{mat}}=200$; and the DART disk-radius and neighborhood settings of Section 3.6), to Scaffold-GS [3], an anchor-based backbone architecturally far from vanilla 3DGS, which voxelizes the point cloud into anchors that each spawn several view-dependent neural Gaussians. DART augments the cloud that seeds these anchors; AGE gates anchor growth.

*Quality.* Under our unified protocol, Ours (Scaffold-GS) is the strongest configuration in the comparison on six of the nine columns of Table 1: Mip-NeRF 360 PSNR (28.07), all three Tanks & Temples metrics (24.46/0.871/0.130), and Deep Blending PSNR and SSIM (30.28/0.913); it is second-best on the remaining three.

*Lift over the reproduced backbone.* Measured against our own Scaffold-GS^∗^ reproduction under the same protocol (Table 6), not the published Scaffold-GS results of Table 1, which come from a different pipeline (Section 3.7), DART + AGE raises Mip-NeRF 360 by +0.37 dB PSNR, +0.012 SSIM, and −0.028 LPIPS, and Tanks & Temples by +0.29 dB, +0.012, and −0.033. On the densely covered Deep Blending the change is marginal and mixed (−0.11 dB PSNR, +0.002 SSIM, −0.012 LPIPS), consistent with Section 5.2: DART’s coverage gains concentrate on sparsely initialized outdoor scenes, while a backbone already dense with anchors has little to gain. LPIPS, the perceptual metric, improves on all three datasets.

*Versus 3D-HGS on the same backbone.* Table 2 places Ours (Scaffold-GS) beside Scaffold-HGS, the half-Gaussian densification of 3D-HGS [26] applied to the same Scaffold-GS backbone, as reported in that paper. On this like-for-like comparison Ours leads eight of the nine metrics (all three Mip-NeRF 360 columns, all three Tanks & Temples metrics, and Deep Blending SSIM and LPIPS) and trails only on Deep Blending PSNR (30.28 vs. 30.36). On a shared backbone, an initialization-and-gating add-on is therefore competitive with, and mostly ahead of, a representation-level change to the Gaussian primitive.

*Anchor growth.* DART enlarges the Scaffold-GS anchor set, with a strongly scene-dependent effect (Table 7). On the already well-covered bicycle and stump the count rises only 12–19%, whereas on the sparsely anchored counter and drjohnson it rises +310% and +106%; the mean is +64% (0.63→1.03M anchors). This growth is consistent with coverage need rather than redundancy: it is largest exactly where the initial Scaffold anchoring is sparsest, counter and drjohnson start from the fewest anchors (Table 7), and negligible where coverage is already adequate (bicycle, stump), so the new anchors fill under-covered regions instead of padding well-covered ones. The growth is productive where it is densest: on counter, the largest-growth scene, Ours (Scaffold-GS) reaches 30.24 PSNR against the Scaffold-GS row’s 29.34 in Table A1, a +0.90 dB gain. Counter and bicycle converge to a similar final count (≈1.15 M) from very different baselines, consistent with DART filling under-covered regions before Scaffold-GS’s voxelization homogenizes anchor density. (These are anchor counts, an order of magnitude below the Gaussian counts of the 3DGS backbone.) As on 3DGS, the growth is DART-driven and partly offset by AGE.

*Multiplier sweep.* Sweeping *m* on Scaffold-GS over the four hard scenes (Table 8) reproduces the 3DGS trend: PSNR saturates by m≈5 while LPIPS continues to fall, and m=10 is best on all three mean metrics (28.04/0.848/0.199). As on 3DGS, this mean trend masks per-scene divergence: the dense *drjohnson* peaks earlier (PSNR 29.90 at m=3 versus 29.82 at m=10) and *stump* peaks at m=5, while the mean is carried by scenes that keep improving, such as *counter* (30.09→30.24). We retain m=10 on both backbones as a single tuning-free setting and accept this small loss of per-scene optimality (Section 5.3).

### 4.6. Qualitative Comparison

**Figure 6:**
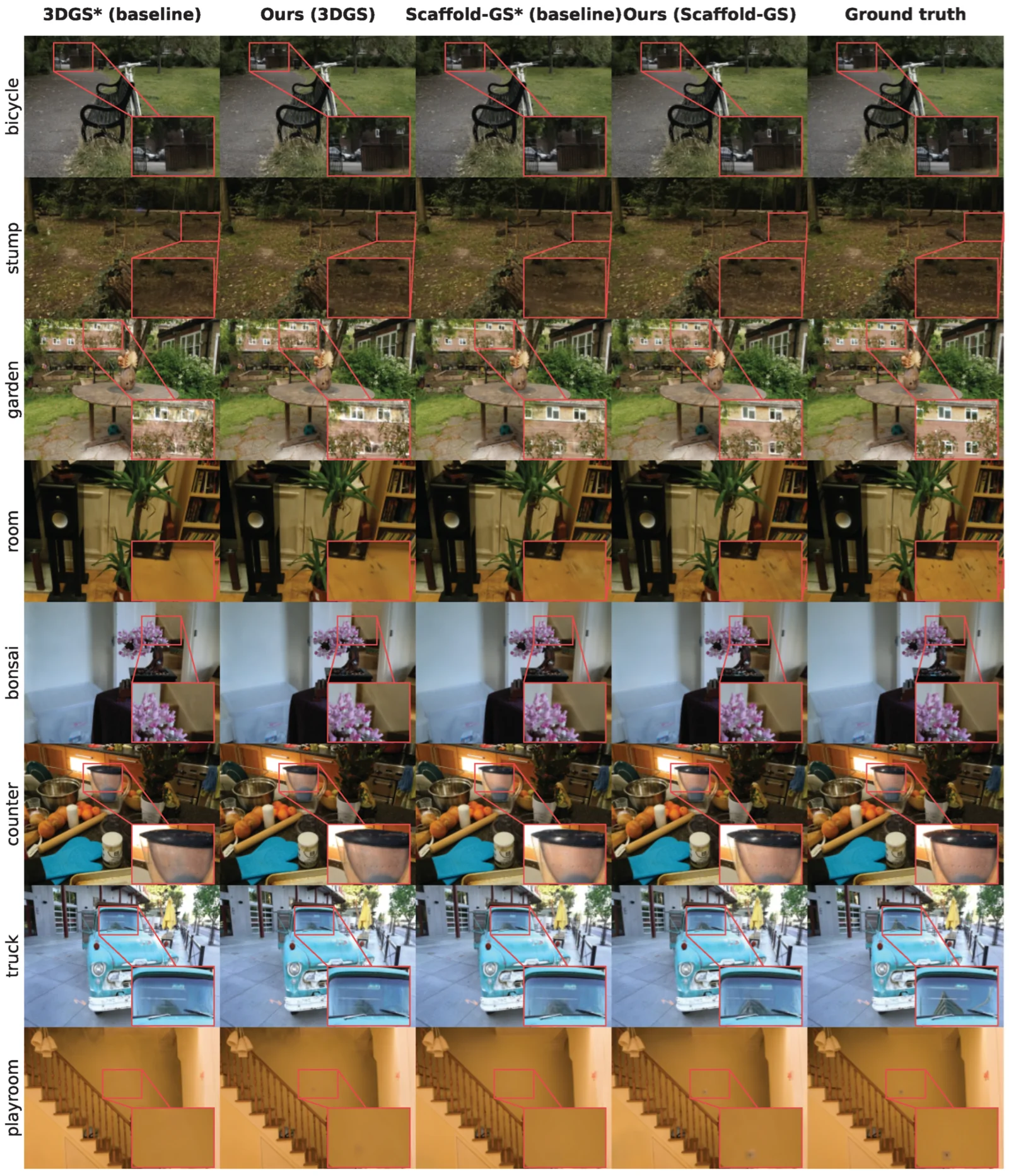
Qualitative comparison on held-out test views. Columns: 3DGS^∗^ (our reproduction), Ours (3DGS), Scaffold-GS^∗^ (our reproduction), Ours (Scaffold-GS), and ground truth. In each row the red box marks a region of interest, enlarged in the inset. Best viewed zoomed in.

Figure 6 shows a qualitative comparison on held-out test views; in each row, the red box marks a region that is enlarged in the side inset. The ordering is perceptual rather than PSNR-based: Ours (Scaffold-GS) ranks first overall in Table 1 and tops most of its columns, and Ours (3DGS) is placed above Scaffold-GS^∗^ (despite the latter’s higher PSNR in several columns) because its LPIPS is lower (0.203 vs. 0.221 on Mip-NeRF 360; Table 1 and Table 6). On the outdoor scenes (bicycle, stump, garden) the benefit appears in the far regions, where the initial SfM points are sparse: our method recovers sharper detail and edges there, consistent with DART adding coverage where the cloud is sparse (Section 5.2). The same effect appears in smooth, weakly textured interiors such as playroom, where feature matching returns few SfM points: Ours (Scaffold-GS) restores faint structure and edges that Scaffold-GS^∗^ misses, with Ours (3DGS) showing the same improvement to a lesser degree. On the truck and counter scenes in particular, Ours (Scaffold-GS) shows the best behavior on specular reflections, expected from Scaffold-GS’s view-dependent neural Gaussians together with the coverage our method adds, rather than from DART alone. The last column, the ground truth, is the reference.

## 5. Discussion

### 5.1. Pipeline Composability

The during-training densification methods of Section 2.3, such as Mini-Splatting-D, intervene on training-loop stages that DART and AGE leave untouched. DART augments the *starting* SfM cloud once, before optimization begins; AGE *gates the eligibility* of existing Gaussians for densification, but uses the published 3DGS criteria once a Gaussian is eligible. Those methods instead condition or reschedule densification during training (Mini-Splatting-D, for instance, periodically replaces Gaussian positions), so their locus of intervention is disjoint from ours, and the two sets of contributions are in principle composable. A combined recipe applying DART for coverage, any of the during-training mechanisms for the densification criterion, and AGE for maturity-gated control of the primitive budget would intervene at three orthogonal stages. We demonstrate one such composition, DART + AGE on the anchor-based Scaffold-GS, in Section 4.5, where the same add-on, unchanged, lifts a backbone architecturally distinct from vanilla 3DGS; combinations with the during-training densification methods above remain future work.

Two recent studies address the growth stage from other directions, and their findings are consistent with ours. Grubert et al. [30] observe that newly created Gaussians evolve differently from their parents because they lack optimizer momentum, and replace the fixed gradient threshold with an exponentially ascending schedule; in their ablation, removing that schedule increases the Gaussian count by about 4% at unchanged PSNR, a pattern similar to AGE’s 8.7–9.6% count reduction at unchanged quality (Table 3). Unlike their iteration-based schedule (Section 2.3), AGE acts on each primitive’s own age. Qiu et al. [35] reduce the model after growth, combining learnable masks, contribution-based pruning, and an opacity update for newly cloned Gaussians, and report a storage reduction of over 30× at a minor cost in rendering quality. AGE instead limits growth before it happens and leaves quality unchanged, at a far smaller reduction; the two address opposite ends of the primitive-budget problem.

### 5.2. Regime of Applicability

The two components have different regimes of usefulness. DART’s gains concentrate on scenes with sparse SfM coverage (typically large outdoor scenes, but also under-covered indoor regions), where its added points most improve perceptual fidelity (Section 4.2); on the sparse outdoor *bicycle* the LPIPS gain reaches −0.044, and even the indoor *counter* improves by −0.024, whereas on the densely reconstructed Deep Blending scene *drjohnson* the initial cloud is already adequate and DART’s effect is small (−0.006). AGE’s count reduction (roughly 9–10%) is, by contrast, largely independent of scene content: it tracks each Gaussian’s maturity rather than scene geometry, and is essentially unchanged whether or not DART augments the cloud (Section 4.2). The method is therefore most useful for sparsely initialized scenes, which are typically large and outdoor; on already densely covered scenes it reduces to a near-neutral initialization with a mild reduction in primitive count. More broadly, this regime is what distinguishes DART from approaches that relax or remove the accurate-SfM requirement itself [15,16], which target settings where reliable SfM is unavailable; DART instead assumes a standard SfM seed and enriches it where it is sparse.

The same regime is consistent with the one comparison in which our Scaffold-GS variant trails on Mip-NeRF 360 PSNR and SSIM under the seven-scene protocol: 3D-GS-MCMC [9] relocates low-opacity Gaussians to occupied regions and is less dependent on the initial point cloud, and it attains the highest seven-scene Mip-NeRF 360 PSNR (29.89 versus 29.75; Table 2).

*Scope of the claims.* Our conclusions hold under the conditions of our experiments: a COLMAP SfM seed from a calibrated multi-view capture, standard ADC without primitive relocation, and the three standard benchmarks. By visual inspection of their SfM seeds, these benchmarks already contain the difficult conditions of real captures: distant outliers (most prominently *drjohnson*, followed by *train* and *playroom*, with points up to 12–20×R from the center), noise around the scene center (*truck* and *train*; 35% of *truck*’s points have an unreliable normal), extremely sparse reconstructions (*stump*, *flowers*, *bicycle*, *treehill*; 32k–54k points), and strong specular reflections (*counter*). SSIM and LPIPS improve on all of these scenes, while PSNR drops slightly on *train* (−0.18 dB) and *treehill* (−0.01 dB). DART uses the distance from a robust scene center as a proxy for SfM sparsity and does not measure local density. The component-level findings (AGE’s 8.7–9.6% count reduction and the dependence of DART’s gain on sparsity) come from a controlled ablation on a subset of scenes.

### 5.3. Limitations

Six limitations bound the scope of our claims. First, the improvements are concentrated in the perceptual metrics (LPIPS and SSIM) rather than in peak PSNR: on the textured, well-covered Tanks & Temples scenes the vanilla-3DGS variant is essentially PSNR-neutral (a 0.04 dB shortfall; Table 1), because DART’s added coverage benefits perceptual fidelity in sparse regions more than it raises a global pixel average. Second, DART enlarges the cloud at initialization (the m=10 multiplier expands the SfM seed roughly tenfold), and the resulting final primitive count is 18.6% above the no-DART baseline before gating (Section 4.2); the two components are therefore coupled by design: DART supplies the quality gain and AGE is required to keep the resulting budget in check, rather than DART being individually beneficial on cost. Third, we deliberately use a single fixed configuration, one multiplier m=10 and one radius schedule, for every scene and dataset, rather than tuning per scene. This trades the best attainable per-scene result for a tuning-free recipe: some scenes peak at a smaller multiplier, most visibly the densely reconstructed *drjohnson*, whose PSNR is higher at m=3 than at the adopted m=10 on both backbones (e.g., 29.90 vs. 29.82 on Scaffold-GS, Table 8, and 29.58 vs. 29.31 on 3DGS, Table 4). We regard this loss of per-scene optimality as an acceptable price for a configuration that transfers unchanged across backbones and scenes. Fourth, DART assumes a locally planar surface and does not validate its replicas against the images; where the PCA normal is unreliable (thin structures, edges, isolated points), replicas may land off the surface. Such normals are common: 10–42% of SfM points per scene have a surface variation $\lambda_{\min}/\left(\lambda_{1}+\lambda_{2}+\lambda_{3}\right)$ above 0.1 (Table A10). This fraction, however, is not associated with a smaller gain (Spearman p>0.26 for all three metrics), and ignoring the normal altogether (isotropic ball, Table 3) costs only 0.12 dB and still exceeds the baseline (27.68 vs. 27.45 dB). Fifth, the radius schedule matters more than its endpoints suggest: applying the upper bound $\rho_{\max}$ uniformly to every point, rather than only to the most peripheral ones, degrades quality below the no-replication baseline and, on *stump*, inflates peak memory to 14.0 GB before pruning removes the misplaced primitives (Section 4.2). The endpoints $\rho_{\min}=0.008$ and $\rho_{\max}=0.04$ were fixed by preliminary experimentation across several scenes and held constant thereafter; we report no sweep over them here, so a scene whose periphery calls for a substantially different scale may fall outside the validated regime. Sixth, the method has a net practical cost. AGE bounds the cost added by DART, recovering about 64% of its added primitives, half of its added peak memory, and a quarter of its added training time, but it does not bring the cost below the unaugmented baseline: relative to 3DGS^∗^, the complete method increases training time by 27.8% (23.0→29.4 min), peak memory by 9.0% (5.11→5.57 GB), and the final Gaussian count by 5.7% on the four ablation scenes (Table 3). The training logs (Figure 5) show that this cost is not concentrated in early training but follows the Gaussian count through densification, and that it is strongly scene-dependent: it is largest on *counter*, whose already dense SfM cloud is replicated tenfold, and vanishes for peak memory on *stump*. Where memory is constrained, m=3 retains most of the PSNR gain of m=10 (Table 4).

## 6. Conclusions

Point-initialized 3D Gaussian Splatting is limited by a sparse, unevenly distributed SfM seed and by a densification cascade that inflates the primitive budget. We addressed both with two backbone-agnostic add-ons: DART, a depth-free and learning-free replication of each SfM point on its local tangent disk with a radius that grows toward the scene periphery, and AGE, which lets a Gaussian split or clone only after it reaches a minimum age. Applied with identical hyperparameters to vanilla 3DGS and Scaffold-GS, they improve both backbones. On Mip-NeRF 360, the 3DGS variant gains 0.30 dB PSNR over the matched baseline, raises SSIM from 0.811 to 0.825, and lowers LPIPS from 0.225 to 0.203, and the Scaffold-GS variant is the strongest configuration on six of the nine dataset–metric columns. Qualitatively, the method recovers sharper detail in sparsely seeded peripheral regions and weakly textured surfaces, while AGE bounds the cost that DART adds. Because both components act outside the rasterizer and the optimizer, they can be carried to other point-initialized pipelines; natural next steps are an in-loop DART that sets the multiplier *m* locally from the accumulated opacity of each voxel, and testing AGE on backbones with strong primitive growth and DART on efficiency-oriented pipelines.

## Figures and Tables

**Table 1:** Main comparison: nine Mip-NeRF 360 scenes, plus Tanks & Temples and Deep Blending (mean across scenes). **Bold** = best in column; underlined = second best. ↑ higher is better, ↓ lower is better. All rows follow the evaluation protocol of Section 3.5. Rows marked ^∗^ are our reproductions; FastGS-Big^∗^ is run with the authors’ per-scene configurations and with one unified configuration (Section 3.7). ^§^ For Scaffold-GS, flowers and treehill (not evaluated in its original paper) are our reproduction. Ours is trained under the compactness-regularized objective of Section 3.4 (whose effect is isolated in Section 4.2).

	Mip-NeRF 360			Tanks & Temples			Deep Blending		
Method	PSNR↑	SSIM↑	LPIPS↓	PSNR↑	SSIM↑	LPIPS↓	PSNR↑	SSIM↑	LPIPS↓
3DGS (2023) [2]	27.20	0.815	0.214	23.15	0.841	0.183	29.41	0.903	0.244
3DGS^∗^	27.38	0.811	0.225	23.69	0.851	0.174	29.76	0.908	0.250
Scaffold-GS (2024) ^§^ [3]	27.52	0.809	0.234	23.96	0.853	0.176	30.21	0.905	0.254
Mini-Splatting-D (2024) [6]	27.51	**0.831**	**0.176**	23.23	0.853	0.141	29.88	0.907	**0.211**
Taming-3DGS (2024) [7]	27.78	0.821	0.209	24.06	0.850	0.170	30.06	0.910	0.240
DashGaussian (2025) [28]	27.73	0.817	0.217	24.00	0.853	0.178	29.65	0.906	0.246
FastGS-Big^∗^ (per-scene config) [27]	27.83	0.819	0.217	24.42	0.857	0.174	30.14	0.911	0.241
FastGS-Big^∗^ (unified config) [27]	27.31	0.806	0.244	23.52	0.840	0.208	30.05	0.909	0.255
Ours (3DGS)	27.68	0.825	0.203	23.65	0.859	0.154	29.95	0.910	0.243
Ours (Scaffold-GS)	**28.07**	0.827	0.193	**24.46**	**0.871**	**0.130**	**30.28**	**0.913**	0.228

**Table 2:** Comparison on the seven-scene Mip-NeRF 360 subset (minus flowers/treehill), plus Tanks & Temples and Deep Blending; unified 3D-HGS protocol. **Bold** = best in column; underlined = second best. ↑ higher is better, ↓ lower is better. ‡ = our row, computed on the same seven scenes.

	Mip-NeRF 360^7^			Tanks & Temples			Deep Blending		
Method	PSNR↑	SSIM↑	LPIPS↓	PSNR↑	SSIM↑	LPIPS↓	PSNR↑	SSIM↑	LPIPS↓
3DGS (2023) [2]	28.88	0.870	0.182	23.60	0.847	0.181	29.41	0.903	0.243
Scaffold-GS (2024) [3]	28.95	0.848	0.220	23.96	0.853	0.177	30.21	0.906	0.254
2D-GS (2024) [23]	28.98	0.867	0.185	23.43	0.845	0.181	29.70	0.902	0.250
FreGS (2024) [25]	27.85	0.826	0.209	23.96	0.841	0.183	29.93	0.904	0.240
GES (2024) [24]	28.69	0.857	0.206	23.35	0.836	0.198	29.68	0.901	0.252
Mip-Splatting (2024) [22]	29.39	0.880	**0.162**	23.75	0.857	0.157	29.46	0.903	0.243
3D-GS-MCMC (2024) [9]	**29.89**	**0.900**	0.190	24.29	0.860	0.190	29.67	0.890	0.320
3D-HGS (2025) [26]	29.66	0.873	0.178	24.45	0.857	0.169	29.76	0.905	0.242
Scaffold-HGS (2025) [26]	29.25	0.867	0.186	24.42	0.859	0.162	**30.36**	0.910	0.240
Ours (3DGS) ‡	29.26	0.880	0.171	23.65	0.859	0.154	29.95	0.910	0.243
Ours (Scaffold-GS) ‡	29.75	0.882	0.163	**24.46**	**0.871**	**0.130**	30.28	**0.913**	**0.228**

**Table 3:** Ablation: rendering quality and cost on the 3DGS backbone; means over the 4 ablation scenes (bicycle, stump, counter, drjohnson). Configurations 1–6 are incremental, each adding one component to the previous row: the regularizer is read both against the baseline (configuration 1 versus 2) and at the full model (5 versus 6), while DART and AGE are read with the regularizer held fixed. Configurations 7–9 are controls that each replace a single geometric design choice inside configuration 6, holding the replica count, the sampling seed, and the objective fixed. #G is the final Gaussian count (millions); time is wall-clock optimization time (minutes) and VRAM is peak allocated memory (GB), both measured on the same GPU (Section 3.6) under identical settings. All nine configurations report measured results. **Bold** marks the best value in a quality column and underline the second best; the cost columns are not ranked.

		Rendering Quality			Cost		
#	Configuration	PSNR↑	SSIM↑	LPIPS↓	#G (M)	Time (min)	VRAM (GB)
*Incremental: each row adds one component*							
1	3DGS^∗^ (baseline)	27.45	0.833	0.228	2.98	23.0	5.11
2	+ Reg	27.53	0.834	0.228	2.91	24.9	5.04
3	+ Reg + DART (m=1) + AGE	27.57	0.834	0.230	2.63	24.2	4.59
4	+ Reg + DART (m=10)	27.75	0.847	**0.203**	3.45	31.8	6.04
5	+ DART (m=10) + AGE (Ours w/o Reg)	27.75	0.847	**0.203**	3.23	29.7	5.68
6	+ Reg + DART + AGE (Ours)	**27.80**	**0.848**	0.204	3.15	29.4	5.57
*Geometry controls: each row replaces one design choice in configuration 6*							
7	constant radius ρ=0.024 (schedule midpoint)	27.69	0.847	0.205	2.99	28.8	5.24
8	constant radius ρ=0.040 (schedule maximum)	26.25	0.797	0.247	2.57	26.7	7.34
9	isotropic ball instead of tangent disk	27.68	0.843	0.205	3.52	30.8	7.16

**Table 4:** Sweep of DART multiplier *m* (AGE fixed at $T_{\mathrm{mat}}=200$). **Bold** = best within this sweep; underlined = second best.

	Bicycle			Stump			Counter			drjohnson			Mean		
Configuration	PSNR↑	SSIM↑	LPIPS↓	PSNR↑	SSIM↑	LPIPS↓	PSNR↑	SSIM↑	LPIPS↓	PSNR↑	SSIM↑	LPIPS↓	PSNR↑	SSIM↑	LPIPS↓
Ours @ m=1	25.16	0.753	0.237	26.68	0.768	0.230	29.00	0.907	0.208	29.43	**0.908**	0.245	27.57	0.834	0.230
Ours @ m=3	25.40	0.771	0.210	26.97	0.781	0.214	29.18	0.913	0.196	**29.58**	**0.908**	0.242	27.78	0.843	0.215
Ours @ m=5	25.48	0.776	0.201	27.02	0.785	0.209	29.24	0.915	0.189	29.37	**0.908**	0.240	27.78	0.846	0.210
Ours @ m=7	25.49	0.778	0.196	**27.08**	**0.787**	0.205	29.28	0.916	0.186	29.31	**0.908**	0.240	27.79	0.847	0.207
Ours @ m=10	**25.50**	**0.781**	**0.190**	27.06	**0.787**	**0.203**	**29.34**	**0.917**	**0.183**	29.31	0.907	**0.238**	**27.80**	**0.848**	**0.204**

**Table 5:** Robustness to random seed (mean ± std over seeds {0,1,2}).

Scene	Method	PSNR↑	SSIM↑	LPIPS↓
*bicycle*	3DGS^∗^ (baseline)	25.135±0.012	0.7532±0.0005	0.2322±0.0008
*bicycle*	Ours (DART + AGE)	25.455±0.010	0.7793±0.0002	0.1902±0.0001
*counter*	3DGS^∗^ (baseline)	29.050±0.019	0.9082±0.0001	0.2035±0.0002
*counter*	Ours (DART + AGE)	29.323±0.011	0.9158±0.0001	0.1809±0.0001

**Table 6:** DART + AGE on Scaffold-GS: lift over our Scaffold-GS^∗^ reproduction. Dataset means under the unified protocol of Section 3.7. Scaffold-GS^∗^ is our reproduction (distinct from the published Scaffold-GS figures of Table 1). Per-scene Ours values appear in Table A1, Table A2, Table A3, Table A4 and Table A5.

	Scaffold-GS^∗^			+ Ours			Δ (Ours − Scaffold-GS^∗^)		
Benchmark	PSNR↑	SSIM↑	LPIPS↓	PSNR↑	SSIM↑	LPIPS↓	PSNR	SSIM	LPIPS
Mip-NeRF 360	27.70	0.815	0.221	28.07	0.827	0.193	+0.37	+0.012	−0.028
Tanks & Temples	24.17	0.859	0.163	24.46	0.871	0.130	+0.29	+0.012	−0.033
Deep Blending	30.39	0.911	0.240	30.28	0.913	0.228	−0.11	+0.002	−0.012

**Table 7:** Adaptive anchor growth under DART (Scaffold-GS backbone). Final anchor count (millions) for four scenes spanning the three datasets.

Scene	Scaffold-GS^∗^ (Baseline)	Scaffold-GS^∗^ + Ours (m=10)	DART Effect
bicycle	0.97 M	1.15 M	+0.18 M (+18.9%)
stump	0.83 M	0.93 M	+0.10 M (+12.1%)
counter	0.28 M	1.15 M	+0.87 M (+310.0%)
drjohnson	0.43 M	0.89 M	+0.46 M (+105.9%)
Mean	0.63 M	1.03 M	+0.40 M (+64.1%)

**Table 8:** DART multiplier *m* on Scaffold-GS (AGE fixed at $T_{\mathrm{mat}}=200$). Four hard scenes (bicycle, stump, counter, drjohnson). **Bold** = best within this sweep; underlined = second best.

	Bicycle			Stump			Counter			drjohnson			Mean		
Configuration	PSNR↑	SSIM↑	LPIPS↓	PSNR↑	SSIM↑	LPIPS↓	PSNR↑	SSIM↑	LPIPS↓	PSNR↑	SSIM↑	LPIPS↓	PSNR↑	SSIM↑	LPIPS↓
Ours @ m=3	25.35	0.772	0.206	26.75	0.777	0.220	29.93	0.919	0.182	**29.90**	0.911	0.233	27.98	0.845	0.210
Ours @ m=5	25.38	0.776	0.197	**26.79**	**0.779**	0.216	30.09	0.921	0.177	29.84	**0.912**	0.230	28.03	0.847	0.205
Ours @ m=7	25.40	0.778	0.191	26.73	0.778	0.213	30.09	0.921	0.176	29.83	0.911	0.227	28.01	0.847	0.202
Ours @ m=10	**25.43**	**0.780**	**0.186**	26.66	0.777	**0.212**	**30.24**	**0.923**	**0.171**	29.82	0.911	**0.225**	**28.04**	**0.848**	**0.199**

## Data Availability

This study uses the publicly available Mip-NeRF 360 [12], Tanks and Temples [13], and Deep Blending [14] benchmark datasets, cited in the manuscript. The DART/AGE implementation is provided as Supplementary Materials accompanying this publication.

## Supplementary Materials

The following supporting information can be downloaded at: https://www.mdpi.com/article/10.3390/jimaging12100483/s1, File S1: Source code of the DART and AGE implementation.

## Notation

$P={\left\{p_{i}\right\}}_{i=1}^{N}$	Input SfM point cloud
$P_{\mathrm{aug}}$	Cloud after DART augmentation
c	Scene center (Weiszfeld L1-median, Equation (1))
*R*	Scene radius (95th-percentile, Equation (3))
$T_{w}$	Weiszfeld iterations
$d_{i}$	$∥p_{i}{-c∥}_{2}$ (Equation (4))
${\hat{d}}_{i}$	Normalized, clipped distance $\mathrm{clip}(d_{i}/R,\;0,1)$ (Equations (4) and (7))
$N_{i}$	$K_{\mathrm{nn}}$ nearest neighbors of $p_{i}$ in P
$n_{i}$	Unit normal at $p_{i}$ (Equation (5))
$\left(e_{i}^{\left(1\right)},e_{i}^{\left(2\right)}\right)$	Tangent basis (Equation (6))
$K_{\mathrm{nn}}$	PCA neighborhood size
$\rho_{\min},\rho_{\max}$	Disk-radius bounds, as fractions of *R*
$\rho_{i}$	Per-point disk radius (Equation (7))
*m*	DART replication multiplier
$p_{i}^{\left(k\right)}$	*k*-th new sample around $p_{i}$ (Equation (9))
$G^{\left(0\right)}$	Initial 3D Gaussian set (after DART-augmented init)
$b_{i}$	Birth iteration of Gaussian *i* (set when *i* is created)
$\nu_{i}\left(t\right)$	Age of Gaussian *i* at iteration *t*: $\nu_{i}=t-b_{i}$ (Equation (10))
$T_{\mathrm{mat}}$	AGE maturity threshold (default 200 iterations)
M(t)	Mature Gaussian set at iteration *t*: $\left\{i:\nu_{i}\left(t\right)\geq T_{\mathrm{mat}}\right\}$
$\Delta_{d}$	Densification cadence (every $\Delta_{d}$ iterations; default 100)
$\tau_{g}$	3DGS gradient threshold for split-or-clone

## Appendix A. Per-Scene Quantitative Results

This appendix provides the per-scene values for the methods reported in Table 1. Rows marked ^∗^ and the Ours rows are measured by us under the protocol of Section 3.6; all other rows reproduce the numbers published in the corresponding original papers. Each table reports one metric, with the nine Mip-NeRF 360 scenes on top and Tanks & Temples, Deep Blending, and the per-dataset means below; the means match Table 1. For Scaffold-GS [3] (marked ^§^), *flowers* and *treehill* are our reproduction, since the original paper evaluates only the other seven Mip-NeRF 360 scenes.

**Table A1:** Per-scene PSNR↑ for all methods of Table 1: Mip-NeRF 360 scenes. **Bold** = best, underlined = second best. ^§^ Scaffold-GS: flowers and treehill are our own reproduction (not evaluated in its original paper); the remaining entries are published values.

Method	Bicycle	Flowers	Garden	Stump	Treehill	Room	Counter	Kitchen	Bonsai
3DGS (2023)	25.25	21.52	27.41	26.55	22.49	30.63	28.70	30.32	31.98
3DGS^∗^	25.18	21.50	27.36	26.31	22.59	31.25	29.02	31.07	32.13
Scaffold-GS (2024) ^§^	24.50	21.42	27.17	26.27	23.08	31.93	29.34	31.30	32.70
Mini-Splatting-D (2024)	**25.55**	21.50	27.67	27.11	22.13	31.41	28.72	31.75	31.72
Taming-3DGS (2024)	25.47	21.76	27.64	26.96	**23.09**	32.12	29.03	31.74	32.22
DashGaussian (2025)	25.31	**21.78**	27.57	**27.17**	22.94	31.81	29.11	31.69	32.15
FastGS-Big^∗^ (per-scene config)	25.23	21.65	27.39	26.98	22.81	31.96	29.45	**32.04**	32.92
FastGS-Big^∗^ (unified config)	25.22	21.43	27.03	26.73	22.59	31.47	28.60	31.21	31.51
Ours (3DGS)	25.50	21.76	27.69	27.06	22.58	31.71	29.34	30.95	32.57
Ours (Scaffold-GS)	25.43	21.36	**27.98**	26.66	23.05	**32.36**	**30.24**	31.77	**33.78**

**Table A2:** Per-scene PSNR↑ (continued): Tanks & Temples, Deep Blending, and per-dataset means (matching Table 1). **Bold** = best, underlined = second best. ^§^ Scaffold-GS: flowers and treehill are our own reproduction (not evaluated in its original paper); the remaining entries are published values.

	Tanks & Temples		Deep Blending		Means		
Method	Truck	Train	drjohnson	Playroom	M360	T&T	DB
3DGS (2023)	25.19	21.10	29.05	29.78	27.20	23.15	29.41
3DGS^∗^	25.30	22.09	29.30	30.22	27.38	23.69	29.76
Scaffold-GS (2024) ^§^	25.77	22.15	29.80	30.62	27.52	23.96	30.21
Mini-Splatting-D (2024)	25.43	21.04	29.32	30.43	27.51	23.23	29.88
Taming-3DGS (2024)	25.90	22.23	29.68	30.44	27.78	24.06	30.06
DashGaussian (2025)	25.80	22.19	29.13	30.17	27.73	24.00	29.65
FastGS-Big^∗^ (per-scene config)	**26.13**	22.72	29.67	30.60	27.83	24.42	30.14
FastGS-Big^∗^ (unified config)	25.44	21.59	29.50	30.60	27.31	23.52	30.05
Ours (3DGS)	25.40	21.91	29.31	30.60	27.68	23.65	29.95
Ours (Scaffold-GS)	26.12	**22.79**	**29.82**	**30.74**	**28.07**	**24.46**	**30.28**

**Table A3:** Per-scene SSIM↑ for all methods of Table 1: Mip-NeRF 360 scenes. **Bold** = best, underlined = second best. Taming-3DGS reports per-scene values to two decimals. ^§^ Scaffold-GS: flowers and treehill are our own reproduction (not evaluated in its original paper); the remaining entries are published values.

Method	Bicycle	Flowers	Garden	Stump	Treehill	Room	Counter	Kitchen	Bonsai
3DGS (2023)	0.771	0.605	0.868	0.775	0.638	0.914	0.905	0.922	0.938
3DGS^∗^	0.754	0.599	0.863	0.762	0.631	0.917	0.908	0.925	0.942
Scaffold-GS (2024) ^§^	0.705	0.594	0.842	0.784	0.643	0.925	0.914	0.928	0.946
Mini-Splatting-D (2024)	**0.798**	**0.642**	**0.878**	**0.804**	0.640	0.928	0.913	**0.934**	0.946
Taming-3DGS (2024)	0.780	0.610	0.870	0.780	0.650	0.920	0.910	0.930	0.940
DashGaussian (2025)	0.763	0.604	0.857	0.783	0.640	0.924	0.911	0.927	0.945
FastGS-Big^∗^ (per-scene config)	0.762	0.614	0.868	0.783	0.628	0.923	0.911	**0.934**	0.948
FastGS-Big^∗^ (unified config)	0.758	0.595	0.839	0.770	0.622	0.914	0.897	0.920	0.937
Ours (3DGS)	0.781	0.619	0.874	0.787	0.643	0.928	0.917	0.928	0.946
Ours (Scaffold-GS)	0.780	0.614	0.877	0.777	**0.656**	**0.933**	**0.923**	0.933	**0.953**

**Table A4:** Per-scene SSIM↑ (continued): Tanks & Temples, Deep Blending, and per-dataset means (matching Table 1). **Bold** = best, underlined = second best. Taming-3DGS reports per-scene values to two decimals. ^§^ Scaffold-GS: flowers and treehill are our own reproduction (not evaluated in its original paper); the remaining entries are published values.

	Tanks & Temples		Deep Blending		Means		
Method	Truck	Train	drjohnson	Playroom	M360	T&T	DB
3DGS (2023)	0.879	0.802	0.897	0.908	0.815	0.841	0.903
3DGS^∗^	0.884	0.819	0.906	0.909	0.811	0.851	0.908
Scaffold-GS (2024) ^§^	0.883	0.822	0.907	0.904	0.809	0.853	0.905
Mini-Splatting-D (2024)	0.890	0.817	0.905	0.908	**0.831**	0.853	0.907
Taming-3DGS (2024)	0.890	0.810	0.910	0.910	0.821	0.850	0.910
DashGaussian (2025)	0.886	0.819	0.903	0.909	0.817	0.853	0.906
FastGS-Big^∗^ (per-scene config)	0.889	0.826	0.907	**0.915**	0.819	0.857	0.911
FastGS-Big^∗^ (unified config)	0.878	0.803	0.905	0.914	0.806	0.840	0.909
Ours (3DGS)	0.889	0.828	0.907	0.914	0.825	0.859	0.910
Ours (Scaffold-GS)	**0.896**	**0.846**	**0.911**	0.914	0.827	**0.871**	**0.913**

**Table A5:** Per-scene LPIPS↓ for all methods of Table 1: Mip-NeRF 360 scenes. **Bold** = best, underlined = second best. Taming-3DGS reports per-scene values to two decimals. ^§^ Scaffold-GS: flowers and treehill are our own reproduction (not evaluated in its original paper); the remaining entries are published values.

Method	Bicycle	Flowers	Garden	Stump	Treehill	Room	Counter	Kitchen	Bonsai
3DGS (2023)	0.205	0.336	0.103	0.210	0.317	0.220	0.204	0.129	0.205
3DGS^∗^	0.232	0.344	0.115	0.230	0.335	0.230	0.204	0.131	0.209
Scaffold-GS (2024) ^§^	0.306	0.347	0.146	0.284	0.322	0.202	0.191	0.126	0.185
Mini-Splatting-D (2024)	**0.158**	**0.255**	**0.090**	**0.169**	**0.262**	0.190	0.172	**0.114**	**0.175**
Taming-3DGS (2024)	0.200	0.340	0.100	0.200	0.310	0.210	0.200	0.120	0.200
DashGaussian (2025)	0.222	0.341	0.123	0.229	0.333	0.205	0.191	0.129	0.180
FastGS-Big^∗^ (per-scene config)	0.232	0.323	0.102	0.226	0.364	0.211	0.193	**0.114**	0.184
FastGS-Big^∗^ (unified config)	0.244	0.347	0.173	0.248	0.357	0.240	0.226	0.145	0.219
Ours (3DGS)	0.190	0.325	0.098	0.203	0.309	0.204	0.183	0.124	0.193
Ours (Scaffold-GS)	0.186	0.307	0.093	0.212	0.284	**0.186**	**0.171**	0.117	0.179

**Table A6:** Per-scene LPIPS↓ (continued): Tanks & Temples, Deep Blending, and per-dataset means (matching Table 1). **Bold** = best, underlined = second best. Taming-3DGS reports per-scene values to two decimals. ^§^ Scaffold-GS: flowers and treehill are our own reproduction (not evaluated in its original paper); the remaining entries are published values.

	Tanks & Temples		Deep Blending		Means		
Method	Truck	Train	drjohnson	Playroom	M360	T&T	DB
3DGS (2023)	0.148	0.218	0.247	0.241	0.214	0.183	0.244
3DGS^∗^	0.146	0.201	0.245	0.254	0.225	0.174	0.250
Scaffold-GS (2024) ^§^	0.147	0.206	0.250	0.258	0.234	0.176	0.254
Mini-Splatting-D (2024)	**0.100**	0.181	**0.218**	**0.204**	**0.176**	0.141	**0.211**
Taming-3DGS (2024)	0.130	0.210	0.240	0.240	0.209	0.170	0.240
DashGaussian (2025)	0.150	0.206	0.250	0.243	0.217	0.178	0.246
FastGS-Big^∗^ (per-scene config)	0.139	0.208	0.245	0.236	0.217	0.174	0.241
FastGS-Big^∗^ (unified config)	0.174	0.241	0.254	0.255	0.244	0.208	0.255
Ours (3DGS)	0.121	0.186	0.238	0.247	0.203	0.154	0.243
Ours (Scaffold-GS)	0.101	**0.159**	0.225	0.231	0.193	**0.130**	0.228

### Appendix A.1. Per-Scene Ablation (Four Scenes)

Per-scene values underlying the means in Table 3 (Section 4.2), for the four ablation scenes (bicycle, stump, counter, drjohnson). Rows correspond to the configurations of Table 3. Within each per-scene table, **bold** marks the best value in a column and underline the second best.

**Table A7:** Per-scene PSNR↑ of the ablation configurations (Table 3).

Configuration	Bicycle	Stump	Counter	drjohnson	Mean
3DGS^∗^ (baseline)	25.18	26.31	29.02	29.30	27.45
+ Reg	25.14	26.56	29.01	29.39	27.53
+ Reg + DART (m=1) + AGE	25.16	26.68	29.00	**29.43**	27.57
+ Reg + DART (m=10)	25.46	27.02	29.33	29.17	27.75
Ours without Reg	25.46	27.03	29.29	29.22	27.75
Ours	**25.50**	**27.06**	**29.34**	29.31	**27.80**

**Table A8:** Per-scene SSIM↑ of the ablation configurations (Table 3).

Configuration	Bicycle	Stump	Counter	drjohnson	Mean
3DGS^∗^ (baseline)	0.754	0.762	0.908	0.906	0.833
+ Reg	0.754	0.766	0.907	0.907	0.834
+ Reg + DART (m=1) + AGE	0.753	0.768	0.907	**0.908**	0.834
+ Reg + DART (m=10)	**0.781**	**0.787**	0.916	0.906	0.847
Ours without Reg	0.779	0.786	0.916	0.907	0.847
Ours	**0.781**	**0.787**	**0.917**	0.907	**0.848**

**Table A9:** Per-scene LPIPS↓ of the ablation configurations (Table 3).

Configuration	Bicycle	Stump	Counter	drjohnson	Mean
3DGS^∗^ (baseline)	0.232	0.230	0.204	0.245	0.228
+ Reg	0.233	0.228	0.207	0.245	0.228
+ Reg + DART (m=1) + AGE	0.237	0.230	0.208	0.245	0.230
+ Reg + DART (m=10)	**0.189**	**0.202**	0.183	0.239	**0.203**
Ours without Reg	0.191	0.203	**0.181**	0.239	**0.203**
Ours	0.190	0.203	0.183	**0.238**	0.204

### Appendix A.2. Reliability of the PCA Normals

**Table A10:** PCA normal reliability and per-scene gain of Ours (3DGS) over 3DGS^∗^. A normal is unreliable when the surface variation of its $K_{\mathrm{nn}}=20$ neighborhood exceeds 0.1. Gains are from Table A1, Table A2, Table A3, Table A4 and Table A5. Spearman correlation between the unreliable fraction and the gain: ρ=+0.16 (PSNR), +0.28 (SSIM), +0.33 (LPIPS); all p>0.26.

Scene	SfM Points	Unreliable Normals	ΔPSNR	ΔSSIM	ΔLPIPS
bicycle	54,275	30.6%	+0.32	+0.027	−0.042
flowers	38,347	41.5%	+0.26	+0.020	−0.019
garden	138,766	26.6%	+0.33	+0.011	−0.017
stump	32,049	9.9%	+0.75	+0.025	−0.027
treehill	52,363	17.4%	−0.01	+0.012	−0.026
room	112,627	30.6%	+0.46	+0.011	−0.026
counter	155,767	28.6%	+0.32	+0.009	−0.021
kitchen	241,367	17.5%	−0.12	+0.003	−0.007
bonsai	206,613	30.2%	+0.44	+0.004	−0.016
truck	136,029	35.1%	+0.10	+0.005	−0.025
train	182,686	26.4%	−0.18	+0.009	−0.015
drjohnson	80,861	9.8%	+0.01	+0.001	−0.007
playroom	37,005	19.7%	+0.38	+0.005	−0.007

## References

[1] Zhang Y., An P., Li Z., Hu S., Liu Q., Yang Y. (2025). A Light Field Guided Optical Synthetic Aperture Imaging System on Master-Slave UAVs. J. Opt. Soc. Am. A.

[2] Kerbl B., Kopanas G., Leimkühler T., Drettakis G. (2023). 3D Gaussian Splatting for Real-Time Radiance Field Rendering. ACM Trans. Graph. (SIGGRAPH).

[3] Lu T., Yu M., Xu L., Xiangli Y., Wang L., Lin D., Dai B. Scaffold-GS: Structured 3D Gaussians for View-Adaptive Rendering. Proceedings of the IEEE/CVF Conference on Computer Vision and Pattern Recognition (CVPR).

[4] Zhang Z., Hu W., Lao Y., He T., Zhao H. Pixel-GS: Density Control with Pixel-aware Gradient for 3D Gaussian Splatting. Proceedings of the European Conference on Computer Vision (ECCV).

[5] Guo Z., Su J., Wang S., Fan J., Zhang J., Han L., Wang P. (2025). GP-GS: Gaussian Processes for Enhanced Gaussian Splatting. arXiv.

[6] Fang G., Wang B. Mini-Splatting: Representing Scenes with a Constrained Number of Gaussians. Proceedings of the European Conference on Computer Vision (ECCV).

[7] Mallick S.S., Goel R., Kerbl B., Steinberger M., Carrasco F.V., Torre F.D.L. (2024). Taming 3DGS: High-Quality Radiance Fields with Limited Resources. Proceedings of the SIGGRAPH Asia 2024 Conference Papers.

[8] Zhou H., Ni Z. Perceptual-GS: Scene-adaptive Perceptual Densification for Gaussian Splatting. Proceedings of the 42nd International Conference on Machine Learning (ICML), PMLR.

[9] Kheradmand S., Rebain D., Sharma G., Sun W., Tseng Y.C., Isack H., Kar A., Tagliasacchi A., Yi K.M. 3D Gaussian Splatting as Markov Chain Monte Carlo. Proceedings of the Advances in Neural Information Processing Systems (NeurIPS).

[10] Cheng K., Long X., Yang K., Yao Y., Yin W., Ma Y., Wang W., Chen X. GaussianPro: 3D Gaussian Splatting with Progressive Propagation. Proceedings of the International Conference on Machine Learning (ICML).

[11] Li Y., Lyu C., Di Y., Zhai G., Lee G.H., Tombari F. GeoGaussian: Geometry-aware Gaussian Splatting for Scene Rendering. Proceedings of the European Conference on Computer Vision (ECCV).

[12] Barron J.T., Mildenhall B., Verbin D., Srinivasan P.P., Hedman P. Mip-NeRF 360: Unbounded Anti-Aliased Neural Radiance Fields. Proceedings of the IEEE/CVF Conference on Computer Vision and Pattern Recognition (CVPR).

[13] Knapitsch A., Park J., Zhou Q.Y., Koltun V. (2017). Tanks and Temples: Benchmarking Large-Scale Scene Reconstruction. ACM Trans. Graph..

[14] Hedman P., Philip J., Price T., Frahm J.M., Drettakis G., Brostow G. (2018). Deep Blending for Free-Viewpoint Image-Based Rendering. ACM Trans. Graph..

[15] Jung J., Han J., An H., Kang J., Park S., Kim S. (2024). Relaxing Accurate Initialization Constraint for 3D Gaussian Splatting. arXiv.

[16] Fu Y., Liu S., Kulkarni A., Kautz J., Efros A.A., Wang X. COLMAP-Free 3D Gaussian Splatting. Proceedings of the IEEE/CVF Conference on Computer Vision and Pattern Recognition (CVPR).

[17] Al-Jaafari M., Abedi F., Yang Y., Liu Q. (2025). Corner Selection and Dual Network Blender for Efficient View Synthesis in Outdoor Scenes. Pattern Recognit..

[18] Lin Z., Huang Y., Yao L. (2024). Three-Dimensional Reconstruction of Indoor Scenes Based on Implicit Neural Representation. J. Imaging.

[19] Fu C., Lu J. (2026). Stereo Gaussian Splatting with Adaptive Scene Depth Estimation for Semantic Mapping. J. Imaging.

[20] Wang X., Xie L., Tang Z., Zhang Q., Zou L., Yang Y., Wu W. (2026). RetinexGS: Enhancing 3D Gaussian Splatting for Low-Light Scenes via Retinex-Guided Decomposition. IEEE Trans. Multimed..

[21] Sun Z., Liu Q., Huang D., Yang Y. (2026). HiGS-3DSC: A Generalizable 3D Gaussian Splatting-based Semantic Communication System for Sparse-View 3D Reconstruction. IEEE Trans. Circuits Syst. Video Technol..

[22] Yu Z., Chen A., Huang B., Sattler T., Geiger A. Mip-Splatting: Alias-free 3D Gaussian Splatting. Proceedings of the IEEE/CVF Conference on Computer Vision and Pattern Recognition (CVPR).

[23] Huang B., Yu Z., Chen A., Geiger A., Gao S. (2024). 2D Gaussian Splatting for Geometrically Accurate Radiance Fields. Proceedings of the SIGGRAPH 2024 Conference Papers.

[24] Hamdi A., Melas-Kyriazi L., Qian G., Mai J., Liu R., Vondrick C., Ghanem B., Vedaldi A. GES: Generalized Exponential Splatting for Efficient Radiance Field Rendering. Proceedings of the IEEE/CVF Conference on Computer Vision and Pattern Recognition (CVPR).

[25] Zhang J., Zhan F., Xu M., Lu S., Xing E. FreGS: 3D Gaussian Splatting with Progressive Frequency Regularization. Proceedings of the IEEE/CVF Conference on Computer Vision and Pattern Recognition (CVPR).

[26] Li H., Liu J., Sznaier M., Camps O. 3D-HGS: 3D Half-Gaussian Splatting. Proceedings of the IEEE/CVF Conference on Computer Vision and Pattern Recognition (CVPR).

[27] Ren S., Wen T., Fang Y., Lu B. FastGS: Training 3D Gaussian Splatting in 100 Seconds. Proceedings of the IEEE/CVF Conference on Computer Vision and Pattern Recognition (CVPR).

[28] Chen Y., Jiang J., Jiang K., Tang X., Li Z., Liu X., Nie Y. DashGaussian: Optimizing 3D Gaussian Splatting in 200 Seconds. Proceedings of the IEEE/CVF Conference on Computer Vision and Pattern Recognition (CVPR).

[29] Liu R., Xu R., Hu Y., Chen M., Feng A. AtomGS: Atomizing Gaussian Splatting for High-Fidelity Radiance Field. Proceedings of the British Machine Vision Conference (BMVC).

[30] Grubert G., Barthel F., Hilsmann A., Eisert P. Improving Adaptive Density Control for 3D Gaussian Splatting. Proceedings of the 20th International Joint Conference on Computer Vision, Imaging and Computer Graphics Theory and Applications (VISAPP).

[31] Foroutan Y., Rebain D., Yi K.M., Tagliasacchi A. (2024). Does Gaussian Splatting need SFM Initialization?. arXiv.

[32] Fan Z., Cong W., Wen K., Wang K., Zhang J., Ding X., Xu D., Ivanovic B., Pavone M., Pavlakos G. (2024). InstantSplat: Sparse-view SfM-free Gaussian Splatting in Seconds. arXiv.

[33] Ying Z., Lv D. (2025). Self-Tuned Two-Stage Point Cloud Reconstruction Framework Combining TPDn and PU-Net. J. Imaging.

[34] Zhang R., Isola P., Efros A.A., Shechtman E., Wang O. The Unreasonable Effectiveness of Deep Features as a Perceptual Metric. Proceedings of the IEEE/CVF Conference on Computer Vision and Pattern Recognition (CVPR).

[35] Qiu S., Wu C., Wan Z., Tong S. (2025). High-Fold 3D Gaussian Splatting Model Pruning Method Assisted by Opacity. Appl. Sci..

